# Integrated Assessment of Soil Contamination and Ecological and Human Health Risks at the Morava Thermal Power Plant Complex: A Three-Year Case Study

**DOI:** 10.3390/toxics14090816

**Published:** 2026-09-13

**Authors:** Nikola Živanović, Vukašin Rončević, Lazar Radulović, Siniša Polovina, Stevan Ćorluka

**Affiliations:** 1 Faculty of Forestry, University of Belgrade, Kneza Višeslava 1, 11000 Belgrade, Serbia; nikola.zivanovic@sfb.bg.ac.rs (N.Ž.); sinisa.polovina@sfb.bg.ac.rs (S.P.); 2 Institute of Chemistry, Technology and Metallurgy, University of Belgrade, Njegoševa 12, 11000 Belgrade, Serbia; vukasin.roncevic@ihtm.bg.ac.rs; 3 Institute for Materials Testing (IMS Institute), Bulevar Vojvode Mišića 43, 11000 Belgrade, Serbia; stevan.corluka@institutims.rs

**Keywords:** coal combustion residues, soil contamination, metals and metalloids, potential ecological risk, human health risk assessment, outdoor worker, reclamation

## Abstract

This three-year case study integrated the results of annual soil-monitoring campaigns conducted in 2022–2024 at 17 predefined locations within and immediately surrounding the Morava Thermal Power Plant Complex, Serbia. We analyzed surface soil samples (0–30 cm) for physical and chemical properties, metals and metalloids, and selected organic pollutants. Contaminant concentrations were evaluated relative to national regulatory thresholds, while potential ecological risk and outdoor worker human health risk were assessed using the potential ecological risk index (RI), Hazard Index (HI), and total carcinogenic risk (TCR). Soil properties and total contaminant concentrations varied considerably among the monitored locations. Regulatory classification identified Ni and Cd as the most widespread concerns. Ni exceeded the applicable limit value at all 17 locations in each campaign, including three remediation-value exceedances in 2022 and one in 2024. Cd exceeded the limit value at 17, 11, and 16 locations in 2022, 2023, and 2024, respectively, without exceeding the remediation value. RI ranged from 210.02 to 963.08, indicating moderate to very high potential ecological risk, and was driven primarily by Cd and Hg. Aggregate HI values ranged from 0.211 to 0.484 and remained below the screening threshold of 1 at all locations. In contrast, TCR ranged from 5.98 × 10^−5^ to 2.12 × 10^−4^ and exceeded the target benchmark of 1 × 10^−6^ at every location in all three campaigns. Arsenic accounted for more than 94% of TCR, while the conservative estimate obtained by applying Cr (VI) toxicity parameters to total Cr represented the principal secondary contribution. The different assessment approaches identified distinct contaminant priorities and therefore provided complementary rather than interchangeable information. The results support continued monitoring, targeted investigation of locations with elevated regulatory or risk indicators, direct chromium-speciation analysis, and integration of soil monitoring with groundwater, drainage-water, bioavailability, and effect-based assessments to support future reclamation and environmental management.

## 1. Introduction

Coal-fired thermal power plants exert substantial environmental pressure through atmospheric emissions and the generation and long-term disposal of coal combustion residues (CCRs), primarily fly ash, bottom ash, and slag, which may contain elevated concentrations of potentially toxic elements [1,2,3]. Potentially toxic elements released through atmospheric emissions and the handling, deposition, and weathering of CCRs may accumulate in surrounding soils, which can act as long-term sinks and secondary sources of contamination [2,4,5,6,7,8]. Following their accumulation in soil, contaminants may persist or be redistributed depending on local physicochemical conditions that regulate their retention, mobility, and potential bioavailability [4,5,9]. Meteorological and hydrological processes, including precipitation, wind, surface runoff, and drainage, may further influence contaminant transport within industrial areas and contribute to differences in contaminant occurrence among individual monitoring locations [6,7,8,10,11,12]. Contaminants retained in soil may subsequently be mobilized and transferred to groundwater and surface water or taken up by plants and other organisms, thereby extending their potential effects beyond soil quality to ecological and human health risks [4,5,13,14,15]. For this reason, an integrated, site-specific assessment of soil contamination associated with coal-fired thermal power plants should consider contaminant concentrations, relevant soil properties, and associated ecological and human health risks [9,16,17].

A major challenge in interpreting soil contamination around coal-fired thermal power plants is that contaminant occurrence and behavior depend not only on the magnitude of pollutant inputs but also on soil properties, including texture, pH, carbonate content, cation exchange capacity, aggregate-size distribution, and water-retention characteristics, which influence contaminant retention, mobility, and potential bioavailability [3,4,5,18]. Accordingly, evaluating contaminant concentrations in relation to relevant soil properties, together with ecological and human health risk indicators, can provide a more comprehensive basis for interpreting soil contamination and its environmental implications [9,19,20]. Contaminants accumulated in soil may contribute to human exposure through incidental soil ingestion, dermal contact, and inhalation of resuspended particles [19,21,22].

Previous studies of soils affected by coal combustion residues have examined contaminant concentrations, transport pathways, and environmental effects from different perspectives, highlighting the value of assessments that integrate these components with soil properties and risk-based indicators [2,4,5]. Previous Serbian studies conducted around coal-fired thermal power plants provide the most relevant regional basis for comparison, although their objectives and analytical scopes differed from those adopted here. Dragović et al. [6] and Ćujić et al. [8] examined selected trace metals around the Nikola Tesla A and B power plants primarily through concentration data, enrichment and contamination indices, multivariate relationships, and spatial or source-oriented analyses. Šajnović et al. [23] evaluated agricultural alluvial soils around the Kostolac power plant using pollution indices and multivariate source analysis, whereas Tanić et al. [24] combined nine heavy metals with ecological indices and health-risk estimates for resident adults and children. These investigations established an essential regional basis for interpreting metal contamination and the combined influence of geogenic and industrial sources, but they did not apply the same combination of regulatory soil-monitoring variables, toxicity-weighted ecological risk, and an occupational soil-exposure scenario within the MTPPC.

The present study includes basic soil chemical properties, water-soluble components, physical and mechanical properties, water-retention characteristics, aggregate-size distribution, total and operationally defined available fractions of metals and metalloids, PAHs, and mineral-oil fractions. These measurements were integrated with national regulatory classification, the Håkanson [25] potential ecological risk index, and the RAIS-based Hazard Index and total carcinogenic risk estimates for the Outdoor Worker scenario. The increment of the study therefore does not lie in proposing a new individual index, but in integrating three annual regulatory monitoring campaigns conducted at the same 17 predefined locations and directly comparing how regulatory, toxicity-weighted ecological, and exposure-based assessments prioritize contaminants within the same monitored system. The resulting dataset also provides a site-specific pre-reclamation reference for future monitoring of the MTPPC, without being interpreted as evidence of a formal temporal trend. The Morava Thermal Power Plant Complex represents a particularly relevant case for an integrated soil-contamination assessment because it combines more than five decades of coal combustion and ash disposal with an environmentally sensitive alluvial setting adjacent to the Great Morava River and near agricultural land and settlements [26]. The long-term operation of the complex is particularly relevant because soil contamination may reflect cumulative and legacy inputs associated with decades of industrial activities and coal-combustion-residue management. Moreover, the ash landfill overlies permeable alluvial deposits and was historically operated largely without impermeable liners or dedicated leachate-control systems, making contaminant retention and potential subsurface transport important site-specific concerns.

The planned reclamation of the ash landfill further increases the environmental and practical relevance of the existing monitoring record. Repeated observations obtained before reclamation provide a valuable screening-level reference against which future changes in soil condition and contaminant occurrence can be evaluated. At the same time, regulatory threshold exceedances alone do not necessarily identify the contaminants of greatest ecological or human-health relevance. Integrating regulatory classification with toxicity-weighted ecological risk and exposure-based human health risk can therefore provide a more informative basis for contaminant prioritization, targeted follow-up investigation, and reclamation planning than any of these approaches considered separately. Accordingly, this three-year case study integrated repeated measurements of soil properties and contaminant concentrations with regulatory, ecological-risk, and outdoor worker health-risk assessments, compared contaminant priorities across the three assessment approaches, identified locations warranting further investigation and provided a pre-reclamation reference for future monitoring.

## 2. Materials and Methods

### 2.1. Study Area

**Figure 1:**
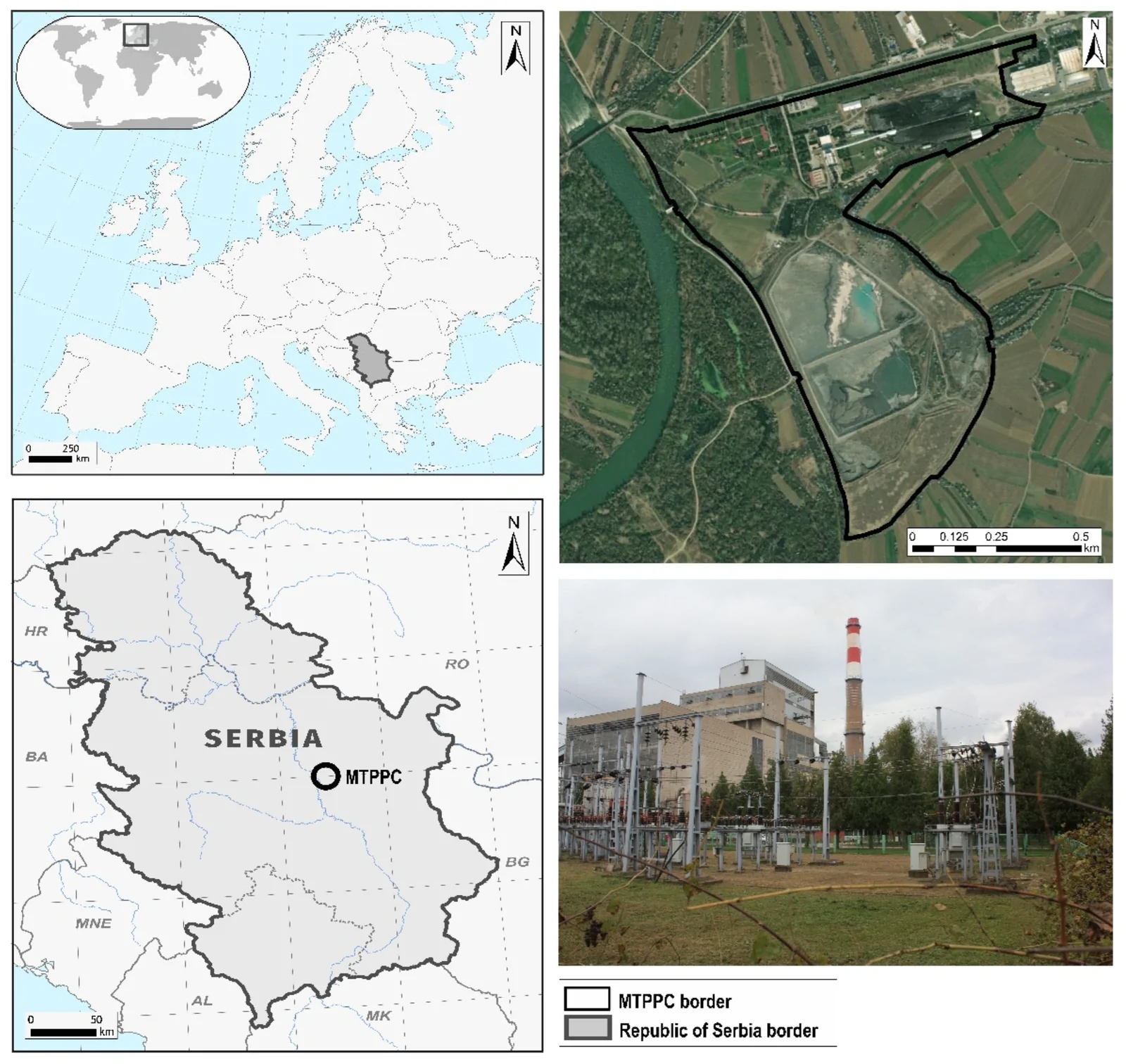
Geographic location and general layout of the MTPPC, including its position within Europe and Serbia, the complex boundary, and a general view of the plant facilities (https://www.eps.rs/lat/tent/Stranice/Proizvodni_kapaciteti.aspx (accessed on 9 September 2026)).

The Morava Thermal Power Plant Complex (MTPPC) has operated since 1969 in the municipality of Svilajnac, central Serbia, primarily to supply electricity during peak-load conditions of the national power system, and it covers approximately 56.4 ha. The complex is located on the right bank of the Great Morava River, near the town of Svilajnac, between 44°12′51.46″ and 44°13′35.76″ N and 21°09′17.85″ and 21°10′14.51″ E. Water from the Great Morava River is used in the thermal power plant cooling system (Figure 1).

The wider area surrounding the MTPPC is characterized by both lowland and hilly landforms. In its northwestern sector, lowland terrain and the alluvial plains of the Resava and Great Morava rivers predominate, whereas the northeastern, eastern, southeastern, and southern sectors are characterized by hilly terrain that gradually transitions into mountainous landscapes. The MTPPC itself is situated on relatively flat and open terrain within the Great Morava River valley, at an elevation of approximately 100 m above sea level.

The climatic characteristics of the MTPPC area were determined using data from the nearest meteorological stations at Bagrdan, RC Petrovac, and Smederevska Palanka, together with data reported for Svilajnac and Kušiljevo by the Republic Hydrometeorological Service of Serbia [27]. The area has a temperate continental climate characterized by pronounced seasonal variations in air temperature, precipitation, and wind conditions. During 1998–2022, the long-term mean daily air temperature was approximately 12.3 °C, while mean daily temperatures were around 0 °C during winter and reached approximately 25 °C during summer. Winds predominantly followed an east–west direction, with easterly winds being the most frequent, accounting for approximately 15% of observations, while wind speeds of 1.5–3.3 m/s occurred most frequently. During 1991–2022, mean annual precipitation was approximately 699 mm. The highest mean monthly precipitation totals, exceeding 70 mm, were recorded in May and June, whereas the lowest totals, approximately 50 mm, occurred in February, March, and November. Mean monthly snowfall totals reached approximately 6–7 cm during the winter months, while snowfall was generally absent from April to October.

The geological framework of the MTPPC area is dominated by Quaternary sediments characteristic of the alluvial plain of the Great Morava River and its tributaries [28]. The principal sedimentary units within the study area are channel and oxbow facies. The channel facies comprise heterogeneous, poorly sorted alluvial deposits of variable thickness, consisting predominantly of gravel, sand, and silty sand. In contrast, the oxbow facies consist of finer sandy-clayey and silty sediments deposited in abandoned river channels [28]. Non-carbonate alluvial deposits predominate in the MTPPC area and constitute the parent material on which the prevailing soils have developed [29]. According to the WRB classification, these soils are predominantly classified as Haplic Fluvisols (Eutric) [30]. They are characterized by pronounced stratification and variable particle-size composition resulting from successive deposition of fluvial material [31], while their properties and ecological functioning are strongly influenced by flooding and groundwater conditions [32,33].

Within the Svilajnac hydrogeological sector of the Great Morava alluvium, the upper cover consists of sandy and clayey silts with a thickness of approximately 4–6 m and hydraulic conductivity values ranging from 10^−7^ to 10^−3^ cm/s. This layer overlies a 2–5 m thick gravelly sandy aquifer with hydraulic conductivity values ranging from approximately 10^−3^ to 10^−1^ cm/s. The aquifer is underlain by relatively impermeable Pliocene clays with hydraulic conductivity below 10^−5^ cm/s [26]. The ash landfill therefore overlies permeable alluvial deposits. Historical ash-disposal operations were largely conducted without impermeable liners or leachate-control systems. Together, these conditions may facilitate infiltration and subsurface contaminant transport [26]. The reported depths and thicknesses describe the local stratigraphic position of the hydrogeological units and should not be interpreted as measurements of groundwater-table depth during the soil-monitoring campaigns. Site-specific groundwater elevations, hydraulic gradients, and seasonal groundwater fluctuations corresponding to the 2022–2024 sampling dates were not available. Therefore, a representative groundwater depth or groundwater-flow direction could not be reliably assigned to the individual monitoring campaigns. Consequently, groundwater-level fluctuations were not included in the analysis of soil-contaminant concentrations.

The wider area surrounding the MTPPC is characterized by a dense network of permanent and intermittent watercourses belonging to the catchments of the Resava and Great Morava rivers. The hydrological regimes of both rivers show pronounced seasonal variability, with the Resava River exhibiting greater discharge fluctuations and the Great Morava River having a comparatively more stable regime [34].

### 2.2. Monitoring Design, Soil Sampling and Laboratory Analyses

The present study was based on existing soil-quality data generated within the regular annual regulatory monitoring program of the MTPPC. The monitoring data were subsequently harmonized, processed, and scientifically interpreted for the present study. Annual soil monitoring was conducted within the MTPPC and its immediate vicinity in 2022, 2023, and 2024 in accordance with the applicable legislation of the Republic of Serbia. The monitoring program comprised soil sampling, sample preparation, laboratory analyses of soil properties and contaminants, and assessment of soil condition and quality.

**Figure 2:**
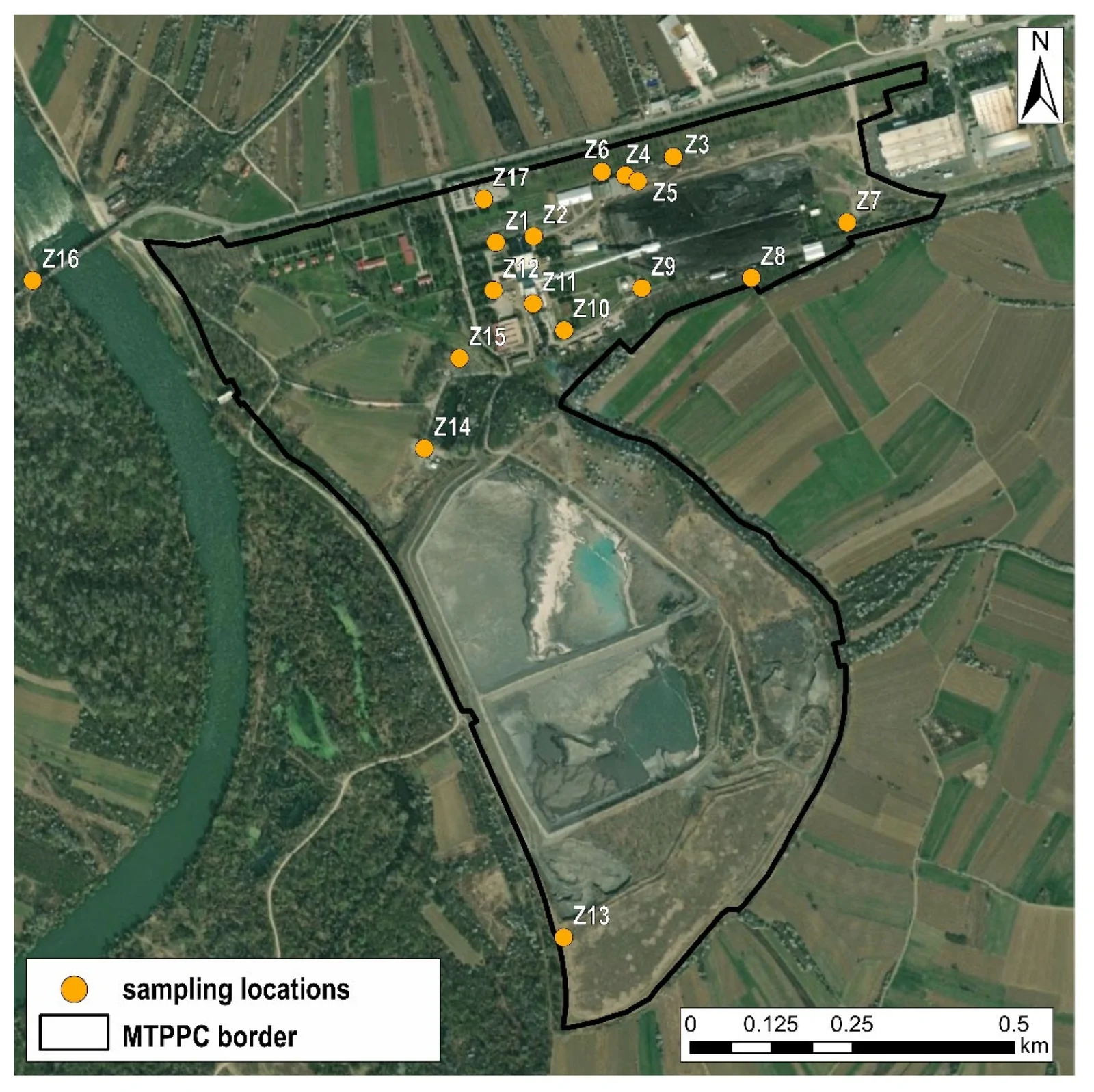
Locations of the 17 predefined soil-monitoring points within and immediately surrounding the MTPPC.

Soil sampling and laboratory analyses were conducted by accredited laboratories in accordance with the Regulation on systematic monitoring of soil status and quality [35], the Regulation on limit values of polluting, harmful and hazardous substances in soil [36], and the Rulebook on permitted quantities of hazardous and harmful substances in soil and irrigation water and methods for their examination [37]. Soil sampling was conducted at the same 17 predefined monitoring locations in 2022, 2023, and 2024 (Figure 2; Table A1). The locations were purposively selected within the monitoring program to cover areas associated with different operational activities and potential contamination sources within the complex and its immediate vicinity. For descriptive interpretation, the locations were assigned to four functional groups based on documented land use, operational function, and proximity to potential contamination sources: electrical and process infrastructure (Z1, Z2, and Z10), storage, fuel, waste, and operational areas (Z3, Z4, Z5, Z6, Z9, Z11, and Z17), coal- and ash-handling and landfill-associated areas (Z7, Z13, Z14, and Z15) and green and peripheral locations (Z8, Z12, and Z16) (Table A1). These groups were defined independently of the measured results and were used only as a descriptive framework for organizing the interpretation of location-level patterns. At each monitoring location, disturbed subsamples were collected from the surface soil layer (0–30 cm) using a soil auger and combined to form one composite sample representative of that location and monitoring campaign. Accordingly, 17 composite surface-soil samples were obtained per campaign, resulting in 51 composite samples across the three campaigns. Separate undisturbed samples were collected for analyses requiring preservation of the natural soil structure. Disturbed and undisturbed soil sampling was performed in accordance with the applicable parts of the ISO 18400 series [38], while soil description followed SRPS EN ISO 25177:2020 [39].

The analytical program included several groups of soil properties and contaminants to support a comprehensive assessment of soil condition and contamination across the monitored network. The analyses comprised basic soil chemical properties, water-soluble ions and components, total concentrations and operationally defined available fractions of metals and metalloids, organic pollutants, aggregate-size distribution, soil water-retention properties, and soil physical and mechanical properties (Table 1). Soil properties were analyzed to characterize soil condition and support the interpretation of contaminant occurrence and behavior, whereas contaminant concentrations provided the basis for regulatory classification and ecological and human health risk assessment at the predefined monitoring locations. The analytical methods applied during the 2022 monitoring campaign are summarized in Table A2, while the corresponding methods used in 2023 and 2024 are presented in Table A3.

### 2.3. Data Processing and Statistical Analysis

#### 2.3.1. Data Harmonization, Treatment, and Descriptive Analysis

Descriptive analyses of soil properties and total concentrations of metals and metalloids were conducted using data from the 17 predefined monitoring locations. Annual observations obtained at the same locations were treated as repeated location-specific observations rather than as independent samples.

Before integration, the analytical procedures, reporting units, and detection limits applied during the three monitoring campaigns were reviewed. Although different standard designations or laboratory procedures were used for some parameters in 2022 and 2023–2024, the corresponding methods were considered comparable with respect to the measured parameter, analytical basis, and reporting unit.

Two complementary data-treatment levels were used. For descriptive characterization of typical conditions at the predefined monitoring locations, one median was calculated for each parameter and each location using the available observations from the 2022, 2023, and 2024 campaigns. Descriptive statistics were subsequently calculated from the resulting 17 location-level values and included the mean, median, standard deviation, minimum, maximum, and coefficient of variation. These location-level medians were also used for exploratory correlation analysis. In contrast, regulatory classification, potential ecological risk assessment, and human health risk assessment were performed separately for each monitoring location and each monitoring campaign using the corresponding annual observations. Thus, annual values were not replaced by location-level medians in any regulatory or risk-based assessment.

For descriptive aggregation, observations reported below the limit of detection were replaced by one-half of the corresponding detection limit (LOD/2) before calculating the location-level median. This procedure was applied to available phosphorus, total nitrogen, ammonium nitrogen, nitrate, and total concentrations of metals and metalloids where censored observations occurred. Parameters for which observations below the detection limit predominated were excluded from the quantitative summaries and assessed qualitatively; accordingly, nitrite was not quantitatively summarized. All measurements of the operationally defined available fractions of Pb, Cd, Hg, As, Cr, Cu, Ni, and Zn were below the corresponding method- and campaign-specific detection limits and were therefore evaluated qualitatively without calculating location-level medians or other descriptive statistics.

For calculation of the individual potential ecological risk factors (E_i_), the overall potential ecological risk index (RI), the Hazard Index (HI), and total carcinogenic risk (TCR), values reported below the limit of detection were conservatively represented by the corresponding full detection limit rather than by LOD/2. The different substitution rules reflected the different purposes of the analyses: LOD/2 was used to reduce upward bias in descriptive summaries, whereas the full LOD was retained in screening-level risk calculations to avoid underestimating potential risk. Regulatory classification, ecological-risk assessment, human health risk assessment, and Spearman correlation analysis were based on measured total concentrations of metals and metalloids.

Campaign-specific proportional-symbol maps were prepared for RI, HI, and TCR using the corresponding values calculated at the predefined monitoring locations in 2022, 2023, and 2024. For each risk indicator, a common symbol-size and color scale was applied across the three campaign maps.

Because of the small number of monitoring locations, potentially non-normal distributions, and tied values associated with censored observations, exploratory associations between selected soil properties and total metal and metalloid concentrations were evaluated using Spearman rank correlation coefficients (ρ). The analysis was performed at the location level using the 17 location-level medians, with each predefined monitoring location represented by one statistical observation. It included total concentrations of Cd, Hg, As, Cu, Pb, Cr, Ni, and Zn and the following soil properties: clay content, cation exchange capacity (CEC), pH measured in H_2_O and KCl, CaCO_3_ content, mean weight diameter (MWD), and geometric mean diameter (GMD). Correlations were calculated using unrounded values, and two-sided *p*-values were obtained. Associations identified on the basis of unadjusted *p*-values were treated as exploratory, hypothesis-generating patterns rather than as confirmatory statistical evidence. The Benjamini–Hochberg procedure was applied across the 56 evaluated correlations to control the false discovery rate, with an adjusted *p*-value < 0.05 considered statistically significant.

Data processing and statistical analyses were performed using Microsoft Excel for Microsoft 365 (Version 2108, Build 14326.20404; Microsoft Corporation, Redmond, WA, USA) and IBM SPSS Statistics (Version 28.0.0.0; IBM Corp., Armonk, NY, USA). Campaign-specific maps were prepared using QGIS (Version 3.20 “Odense”; QGIS Development Team).

#### 2.3.2. Operational and Environmental Context of the MTPPC

Annual operational and environmental data for the MTPPC were compiled from the EPS annual environmental reports for 2022, 2023, and 2024. The extracted indicators included gross and net electricity generation, coal and fuel-oil consumption, and reported annual emissions of particulate matter, SO_2_, NO_x_, and CO_2_. The indicators were summarized descriptively and are presented in Table A4.

These data were used to characterize annual plant operating intensity and to provide contextual information for interpreting contaminant exceedances and risk indicators during the soil-monitoring period. Because the indicators were available only as plant-level annual totals and were neither temporally matched to the soil-sampling dates nor spatially resolved among the monitoring locations, they were not used as predictors of soil contaminant concentrations or as evidence of causal relationships.

#### 2.3.3. Soil Aggregate-Size Distribution Indices

Based on the measured aggregate-size distribution across the fractions >10, 10–5, 5–3, 3–2, 2–1, 1–0.5, 0.5–0.25, and <0.25 mm, two indices were calculated to characterize soil aggregation: mean weight diameter (MWD) and geometric mean diameter (GMD). The MWD was calculated as (Equation (1)):

MWD = Σ(x_i_ × w_i_) (1)

where

x_i_—the mean diameter of each aggregate size fraction (mm), and

w_i_—the mass fraction of the corresponding aggregate size class (dimensionless).

The geometric mean diameter (GMD) was calculated as (Equation (2)):

GMD = exp[Σ(w_i_ × ln x_i_)] (2)

where

x_i_—the mean diameter of each aggregate size fraction (mm), and

w_i_—the mass fraction of the corresponding aggregate size class (dimensionless).

Higher MWD and GMD values indicate an aggregate-size distribution weighted toward larger aggregates, whereas lower values indicate a greater relative contribution of finer aggregate fractions. These indices were interpreted as measures of aggregate-size distribution rather than as direct measures of aggregate stability.

#### 2.3.4. Classification of Contaminant Concentrations Relative to Regulatory Threshold Values

Total concentrations of metals and metalloids and measured concentrations of polycyclic aromatic hydrocarbons (PAHs) and mineral oil fractions (C_6_–C_40_) were classified separately for each predefined monitoring location and each of the 2022, 2023, and 2024 monitoring campaigns. The classification was performed relative to the applicable limit and remediation values, corrected for the measured clay and organic-matter contents as prescribed by the Regulation on limit values of polluting, harmful and hazardous substances in soil [35]. Annual observations were not aggregated before regulatory classification.

Three regulatory categories were applied: at or below the applicable limit value, above the limit value but at or below the remediation value, and above the remediation value. These categories were presented in green, orange, and red, respectively. For each contaminant and monitoring campaign, the number of monitoring locations assigned to each regulatory category was summarized descriptively. The campaign-specific classifications were evaluated jointly to describe the occurrence and frequency of regulatory exceedances within the monitored network during the 2022–2024 period.

#### 2.3.5. Potential Ecological Risk Assessment of Metals and Metalloids

The potential ecological risk index (RI) developed by [25] was applied as a screening-level method for assessing the potential ecological risk associated with Cd, Hg, As, Pb, Cu, Ni, Cr, and Zn. The assessment was based on measured total element concentrations, reference background concentrations, and element-specific toxic-response factors. The individual potential ecological risk factor (E_i_) was calculated as follows:

E_i_ = T_i_ × (C_i_/C_ref,i_)

where

E_i_—the individual potential ecological risk factor for element i (dimensionless),

T_i_—the toxic-response factor for element i (dimensionless),

C_i_—the measured total concentration of element i in soil (mg/kg), and

C_ref,i_—the reference background concentration of element i (mg/kg).

The overall potential ecological risk index (RI) was calculated as the sum of the individual potential ecological risk factors:

RI = ΣE_i_

The toxic-response factors (T_i_) were adopted from [25], while the reference background concentrations (C_ref,i_) were obtained from geochemical maps and relevant literature [40] (Table 2). The index was calculated using the total concentrations of the analyzed elements. For concentrations reported below the limit of detection, the corresponding detection limit was conservatively used in the calculations.

The classification criteria for the individual potential ecological risk factor (E_i_) and the overall potential ecological risk index (RI) are presented in Table 3 and Table 4, respectively. The $E_{i}$ value represents the potential ecological risk associated with an individual element, whereas RI represents the cumulative potential ecological risk obtained by summing the E_i_ values of all elements included in the assessment. Consequently, the numerical classification thresholds applied to E_i_ and RI refer to different assessment levels and are not directly comparable.

The individual potential ecological risk factors (E_i_) and the overall potential ecological risk index (RI) were calculated separately for each predefined monitoring location and each of the 2022, 2023, and 2024 monitoring campaigns. Each campaign-specific RI value was classified independently according to the criteria presented in Table 4.

All three campaign-specific RI values were retained for each monitoring location to describe ecological-risk levels during the 2022–2024 period without obscuring individual high-risk observations. The assessment considered campaign-specific RI ranges and categories, individual high-risk observations, and element-specific contributions to the overall RI.

#### 2.3.6. Human Health Risk Assessment

A screening-level human health risk assessment associated with soil exposure was performed using the site-specific risk calculator within the Risk Assessment Information System (RAIS) [42]. The assessment was conducted for the soil medium using the Outdoor Worker scenario, measured site-specific contaminant concentrations, selected site-specific soil and meteorological parameters, and standard RAIS exposure factors; the 500-acre source area corresponded to the predefined assessment zone encompassing the complete sampling network. The soil and exposure parameters used in the calculations are presented in Table A5. Toxicological parameters for the evaluated contaminants, including reference doses (RfDs), reference concentrations (RfCs), slope factors (SFs), and inhalation unit risks (IURs), were obtained from the RAIS database.

Because chemical speciation was not determined, the chemical forms selected in RAIS were used as screening toxicity surrogates; in particular, total Cr was conservatively evaluated using Cr(VI) toxicity parameters, and the resulting Cr-related estimates should not be interpreted as evidence of actual Cr(VI) occurrence.

The outputs retained for analysis were the Hazard Index (HI) for non-carcinogenic effects and the RAIS Total Risk output, hereafter referred to as total carcinogenic risk (TCR). The aggregate HI and TCR included contributions from incidental soil ingestion, dermal contact, and inhalation of resuspended soil particles. HI and TCR were calculated separately for each predefined monitoring location and each of the 2022, 2023, and 2024 monitoring campaigns. HI values were evaluated relative to the screening threshold of 1, while TCR values were evaluated relative to the target risk benchmark of 1 × 10^−6^.

For each monitoring location and campaign, the relative contribution of an individual contaminant to HI was calculated by dividing its contaminant-specific contribution by the corresponding aggregate HI and expressing the result as a percentage. The relative contribution to TCR was calculated analogously by dividing the contaminant-specific carcinogenic risk by the corresponding aggregate TCR.

For the contributor-frequency analysis, a contaminant was classified as a screening-relevant contributor to non-carcinogenic risk at a monitoring location when its contaminant-specific contribution to HI was ≥0.1. A contaminant was classified as a screening-relevant contributor to carcinogenic risk when its contaminant-specific carcinogenic risk was ≥1 × 10^−6^. The number of monitoring locations meeting the corresponding criterion was determined separately for each contaminant and monitoring campaign. These operational criteria were used only to summarize the location-level frequency of contaminant-specific contributions and did not replace the aggregate screening benchmarks of HI = 1 and TCR = 1 × 10^−6^.

#### 2.3.7. Overview of the Monitoring and Assessment Approaches

The repeated monitoring design and the complementary assessment approaches applied in this study are summarized in Table 5. Each approach evaluates a different aspect of soil contamination or risk and is subject to specific assumptions and limitations. The results were therefore interpreted jointly rather than as interchangeable measures of environmental condition or risk.

## 3. Results

### 3.1. Soil Properties and Total Metal and Metalloid Concentrations Based on Location-Level Medians

Location-level median values were calculated from the available annual observations recorded during the 2022–2024 monitoring campaigns, as described in Section 2.3.1. Descriptive statistics calculated from the resulting 17 location-level median values are presented for soil physical, water-retention, mechanical, and aggregate-size properties in Table 6; for soil chemical properties and water-soluble components in Table 7; and for total concentrations of metals and metalloids in Table 8. The results characterize typical conditions recorded at the monitored locations and should not be interpreted as temporal trends or as spatially continuous estimates for the entire MTPPC area.

#### 3.1.1. Soil Physical, Water-Retention, Mechanical, and Aggregate-Size Properties

The location-level median values showed different degrees of variation in soil physical, water-retention, mechanical, and aggregate-size properties among the 17 predefined monitoring locations (Table 6). Clay content ranged from 11.00 to 47.60%, with a mean of 26.96% and a coefficient of variation of 43.73%. In contrast, particle density was comparatively uniform, ranging from 2.64 to 2.74 g/cm^3^ and showing the lowest relative variability among the analyzed properties (CV = 1.10%). Total porosity ranged from 37.24 to 57.54%, with a mean of 51.76% and comparatively limited variation among locations (CV = 10.00%).

Water retention at field capacity ranged from 18.53 to 48.33%, with a mean of 34.62%, whereas water retention at the wilting point ranged from 6.82 to 30.13%, with a mean of 17.63%. Relative variation was greater for water retention at the wilting point (CV = 43.62%) than for water retention at field capacity (CV = 30.20%). Plant-available and readily available water ranged from 6.72 to 24.46%, with a mean of 17.07% and a coefficient of variation of 27.53%.

Bulk density ranged from 1.12 to 1.73 g/cm^3^, with a mean of 1.30 g/cm^3^ and comparatively moderate variation among locations (CV = 12.00%). Penetration resistance showed greater relative variability, ranging from 0.04 to 0.24 MPa, with a mean of 0.14 MPa and a coefficient of variation of 46.70%.

The aggregate-size indices also differed markedly among the monitoring locations. Mean weight diameter ranged from 1.72 to 7.69 mm, with a mean of 4.63 mm and a coefficient of variation of 38.14%. Geometric mean diameter ranged from 0.72 to 5.02 mm, with a mean of 2.63 mm and the highest coefficient of variation among the properties presented in Table 6 (CV = 54.16%). Moisture content at 105 °C, based on the available 2023 and 2024 observations, ranged from 2.18 to 5.86%, with a mean of 3.64% and a coefficient of variation of 22.33%.

#### 3.1.2. Soil Chemical Properties and Water-Soluble Components

The location-level median values of soil chemical properties and water-soluble components also showed different degrees of variation among the predefined monitoring locations (Table 7). Soil pH showed comparatively limited variation. The pH measured in H_2_O ranged from 6.75 to 8.13, with a mean of 7.65 and a coefficient of variation of 4.37%, whereas pH measured in KCl ranged from 5.82 to 7.67, with a mean of 7.09 and a coefficient of variation of 6.29%. Electrical conductivity ranged from 0.23 to 1.52 mS/cm, with a mean of 0.43 mS/cm and substantially greater relative variation among locations (CV = 69.36%).

CaCO_3_ content ranged from 0.19 to 4.91%, with a mean of 1.62% and a median of 1.41%. Cation exchange capacity ranged from 10.18 to 77.00 cmol(+)/kg. Its mean value of 21.23 cmol(+)/kg was higher than the median of 16.66 cmol(+)/kg because of an elevated value at one monitoring location. The coefficients of variation for CaCO_3_ and CEC were 86.76 and 71.91%, respectively, indicating substantial location-specific variation.

Total nitrogen ranged from 0.05 to 0.43%, with a mean of 0.18% and a median of 0.16%. Available phosphorus showed the greatest relative variability among the chemical properties, ranging from 0.50 to 32.80 mg P_2_O_5_/100 g. Its mean value was 5.44 mg P_2_O_5_/100 g, whereas the median was 0.81 mg P_2_O_5_/100 g, indicating a right-skewed distribution influenced by elevated values at a limited number of locations. Available potassium ranged from 9.60 to 55.18 mg K_2_O/100 g, with a mean of 31.49 mg K_2_O/100 g and a median of 32.41 mg K_2_O/100 g.

Among the water-soluble components, ammonium nitrogen ranged from 0.25 to 15.70 mg/kg, with a mean of 7.12 mg/kg. Water-soluble calcium showed pronounced location-specific variation, ranging from 38.10 to 771.50 mg/kg. Its mean value of 131.45 mg/kg was considerably higher than the median of 89.70 mg/kg because of an elevated value at one location. Water-soluble magnesium ranged from 10.34 to 56.92 mg/kg, with a mean of 22.66 mg/kg and a median of 20.14 mg/kg. Nitrate concentrations ranged from 0.50 to 9.45 mg/kg, with nearly identical mean and median values of 4.29 and 4.30 mg/kg, respectively. Nitrite was not quantitatively summarized because observations below the limit of detection predominated.

#### 3.1.3. Total Concentrations of Metals and Metalloids

The location-level median total concentrations of metals and metalloids showed different degrees of variation among the 17 predefined monitoring locations (Table 8). Cadmium concentrations ranged from 0.40 to 3.50 mg/kg, with a mean of 2.69 mg/kg and a median of 2.80 mg/kg. Mercury concentrations ranged from 0.125 to 0.60 mg/kg, with a mean of 0.29 mg/kg and a median of 0.27 mg/kg. Mercury showed comparatively high relative variation among locations (CV = 57.29%). However, the distributions of Cd and Hg should be interpreted cautiously because several annual observations were reported below the limit of detection and were represented by LOD/2 before the location-level medians were calculated.

Arsenic concentrations ranged from 12.54 to 23.55 mg/kg, with a mean of 17.18 mg/kg and a median of 16.65 mg/kg. Lead concentrations ranged from 49.60 to 137.41 mg/kg, with nearly identical mean and median values of 87.51 and 87.60 mg/kg, respectively. Nickel ranged from 95.34 to 174.14 mg/kg, with a mean of 137.32 mg/kg and a median of 135.50 mg/kg. Zinc concentrations ranged from 72.60 to 142.41 mg/kg, with a mean of 114.34 mg/kg and a median of 118.63 mg/kg. Arsenic, nickel, and zinc showed comparatively lower relative variation, with coefficients of variation of approximately 19–21%.

Copper and chromium showed the greatest absolute differences among locations. Copper concentrations ranged from 19.82 to 120.18 mg/kg, with a mean of 39.22 mg/kg and a median of 34.20 mg/kg. The maximum copper concentration was recorded at Z5. The difference between the mean and median, together with a coefficient of variation of 59.72%, indicates that the distribution was influenced by elevated concentrations at a limited number of locations. Chromium concentrations ranged from 74.72 to 321.47 mg/kg, with a mean of 122.59 mg/kg and a median of 106.30 mg/kg. The maximum chromium concentration was recorded at Z4, and its coefficient of variation was 49.06%.

All measurements of the operationally defined available fractions of Pb, Cd, Hg, As, Cr, Cu, Ni, and Zn were below the corresponding method- and campaign-specific limits of detection. Consequently, location-level medians and other quantitative descriptive statistics were not calculated for these fractions. These results indicate that the extracted fractions were below the detection capability of the applied analytical methods and should not be interpreted as zero concentrations or as evidence of the complete absence of potentially available forms.

### 3.2. Regulatory Exceedances During the 2022–2024 Monitoring Period

**Figure 3:**
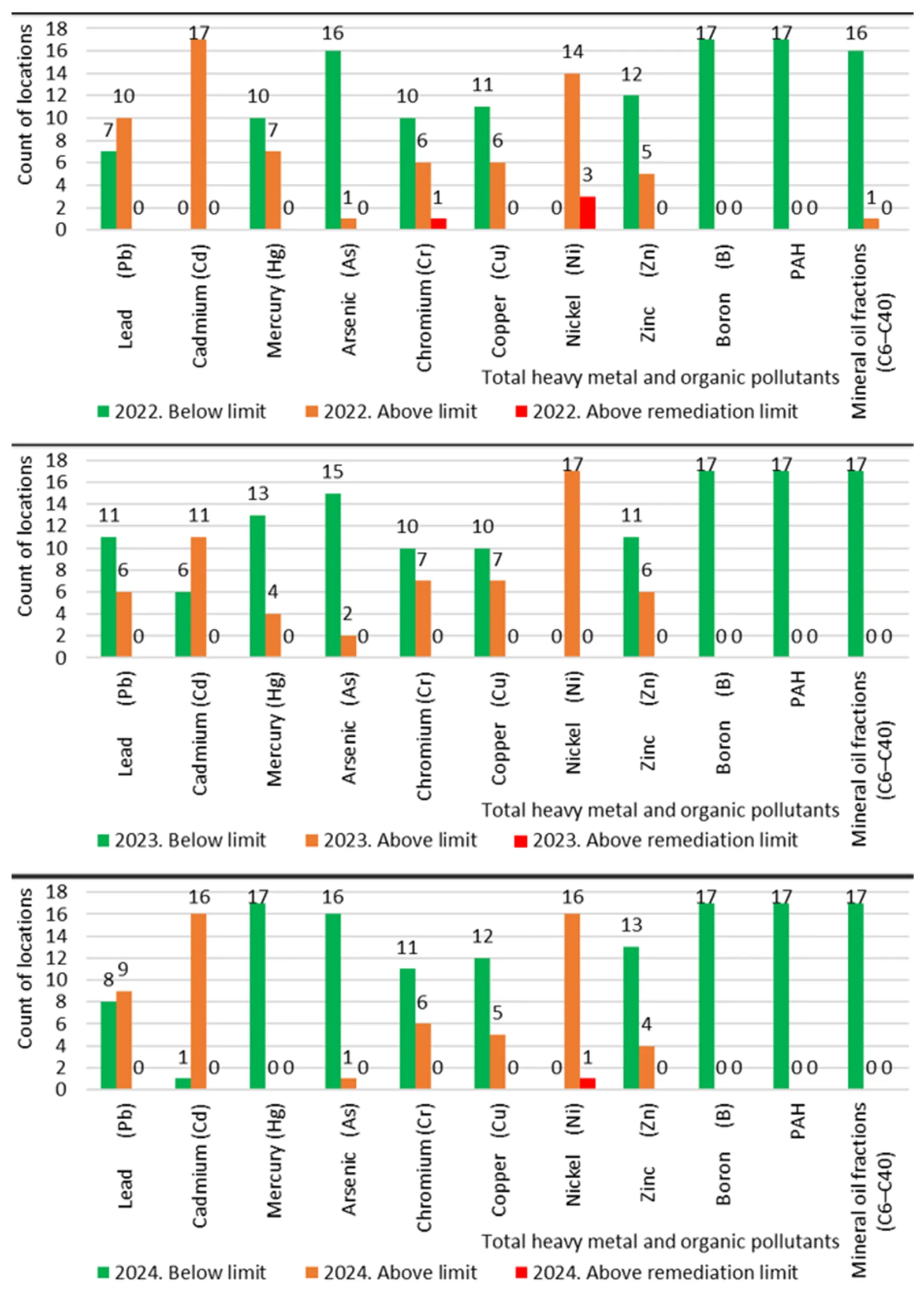
Number of monitoring locations classified relative to the applicable regulatory limit and remediation values for total concentrations of metals and metalloids and selected organic pollutants during the 2022, 2023, and 2024 monitoring campaigns.

Regulatory classification across the three monitoring campaigns was used to describe contaminant exceedances within the monitored network during the 2022–2024 period (Figure 3). Overall, most contaminant–location observations were classified at or below the corresponding regulatory limit values. Nevertheless, exceedances were recorded during multiple campaigns for several metals and metalloids, particularly Cd and Ni.

Cadmium exceedances were recorded in all three monitoring campaigns. The applicable limit value was exceeded at all 17 monitoring locations in 2022, at 11 locations in 2023, and at 16 locations in 2024. No Cd concentration exceeded the remediation value. The occurrence of Cd exceedances in each campaign therefore indicates a widespread regulatory concern within the monitored network.

Nickel showed the most extensive regulatory non-compliance. All 17 monitoring locations exceeded the applicable limit value in each of the three campaigns. In 2022, 14 locations were classified above the limit value but at or below the remediation value, while three locations exceeded the remediation value. In 2023, all 17 locations were above the limit value but at or below the remediation value. In 2024, 16 locations were above the limit value but at or below the remediation value, while one location exceeded the remediation value. Thus, Ni exceedances were consistently present throughout the entire monitoring period, including occasional exceedances of the remediation value.

Exceedances of Pb, Cr, Cu, and Zn were also recorded across all three monitoring campaigns, although they affected fewer locations than Cd and Ni. Lead exceeded the applicable limit value at 10 locations in 2022, six locations in 2023, and nine locations in 2024. Chromium exceedances were recorded at seven locations in 2022, including one location above the remediation value, at seven locations in 2023, and at six locations in 2024. Copper exceeded the limit value at six, seven, and five locations in 2022, 2023, and 2024, respectively, while Zn exceedances were recorded at five, six, and four locations. No remediation-value exceedances were recorded for Pb, Cu, or Zn. These findings indicate more spatially limited regulatory exceedances of Pb, Cr, Cu, and Zn within the monitored network.

Arsenic exceedances occurred at a small number of locations in each campaign, with one exceedance in 2022, two in 2023, and one in 2024. Mercury exceeded the applicable limit value at seven locations in 2022 and four locations in 2023, whereas all 17 locations were classified at or below the limit value in 2024. No remediation-value exceedances were recorded for As or Hg. Accordingly, As exceedances were isolated, while Hg exceedances occurred during two of the three monitoring campaigns.

PAHs were classified at or below their applicable limit values at all 17 monitoring locations in each of the three campaigns. Mineral oil fractions (C6–C40) were also at or below the applicable limit value at all locations in 2023 and 2024, while one exceedance was recorded in 2022. PAHs therefore showed no regulatory exceedances, whereas the mineral-oil exceedance was isolated to one location in 2022. During the same period, gross electricity generation decreased from 616.50 GWh in 2022 to 391.00 GWh in 2023 and 304.10 GWh in 2024, while coal consumption decreased from 850,864 to 531,072 and 396,293 t year^−1^, respectively (Table A4). In contrast, the number of monitoring locations exceeding the applicable Cd limit value decreased from 17 in 2022 to 11 in 2023 and subsequently increased to 16 in 2024. Thus, the Cd regulatory-exceedance pattern did not parallel the monotonic reduction in plant operating intensity. This comparison is descriptive only: the annual operational totals were not matched to the soil-sampling dates or individual monitoring locations and were not analyzed as predictors of Cd concentrations. Overall, Cd and Ni represented the most widespread regulatory concerns during the 2022–2024 monitoring period. Exceedances of Pb, Cr, Cu, and Zn were recorded in all three campaigns but affected a smaller proportion of the monitored network. Arsenic and Hg exceedances were more limited, whereas B, PAHs, were at or below their applicable limit values at all locations in all three campaigns, and mineral oil fractions exceeded the limit value at one location in 2022 only. These exceedances were recorded during a period characterized by decreasing electricity generation and coal and fuel-oil consumption, together with overall reductions in reported atmospheric emissions (Table A4). The correspondence between the operating conditions and the observed contaminant patterns is considered further in the Discussion.

### 3.3. Ecological Risk Levels Based on RI

**Figure 4:**
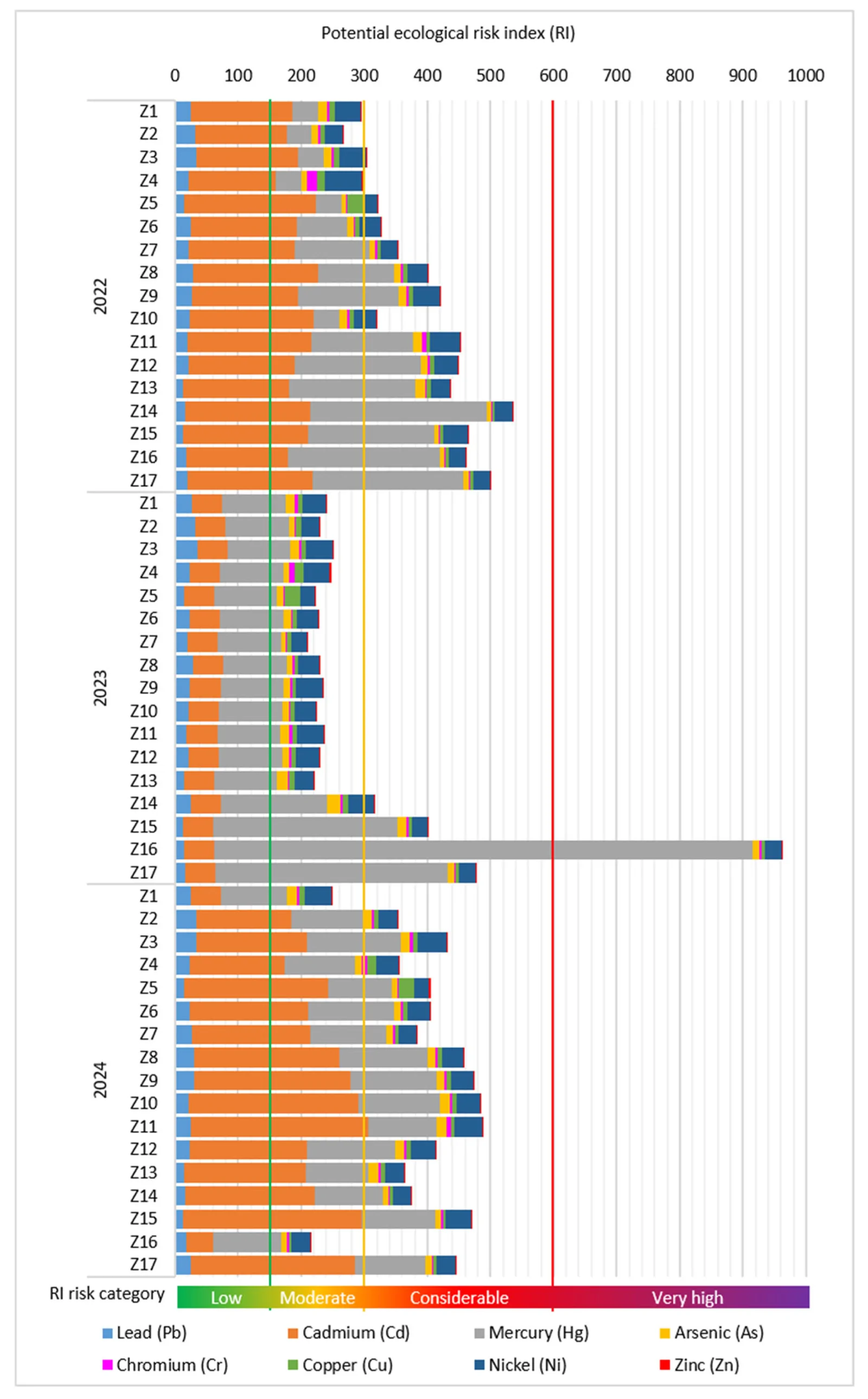
Campaign-specific contributions of individual elements (E_i_) to the overall potential ecological risk index (RI) at the 17 predefined monitoring locations during 2022–2024. The total length of each stacked bar represents the overall RI, while the colored segments represent the element-specific contributions. Vertical reference lines indicate the RI classification thresholds of 150, 300, and 600.

The potential ecological risk index (RI) ranged from 268.03 to 535.14 in 2022, from 210.02 to 963.08 in 2023, and from 215.37 to 487.92 in 2024. No low-risk RI values were recorded during the monitoring period. Moderate and considerable ecological risk occurred in all three campaigns, while the only very-high-risk value was recorded at Z16 in 2023 (RI = 963.08). The value recorded at Z16 in 2023 was substantially higher than the values recorded at the same location in 2022 and 2024 (Figure 4).

The distribution of ecological-risk categories differed among the three monitoring campaigns. In 2022, 14 of the 17 monitoring locations were classified as having considerable ecological risk and three as having moderate risk. In 2023, moderate ecological risk predominated at 13 locations, three locations were classified as having considerable risk, and one location had very high risk. In 2024, considerable ecological risk was recorded at 15 locations, while the remaining two locations were classified as having moderate risk.

**Figure 5:**
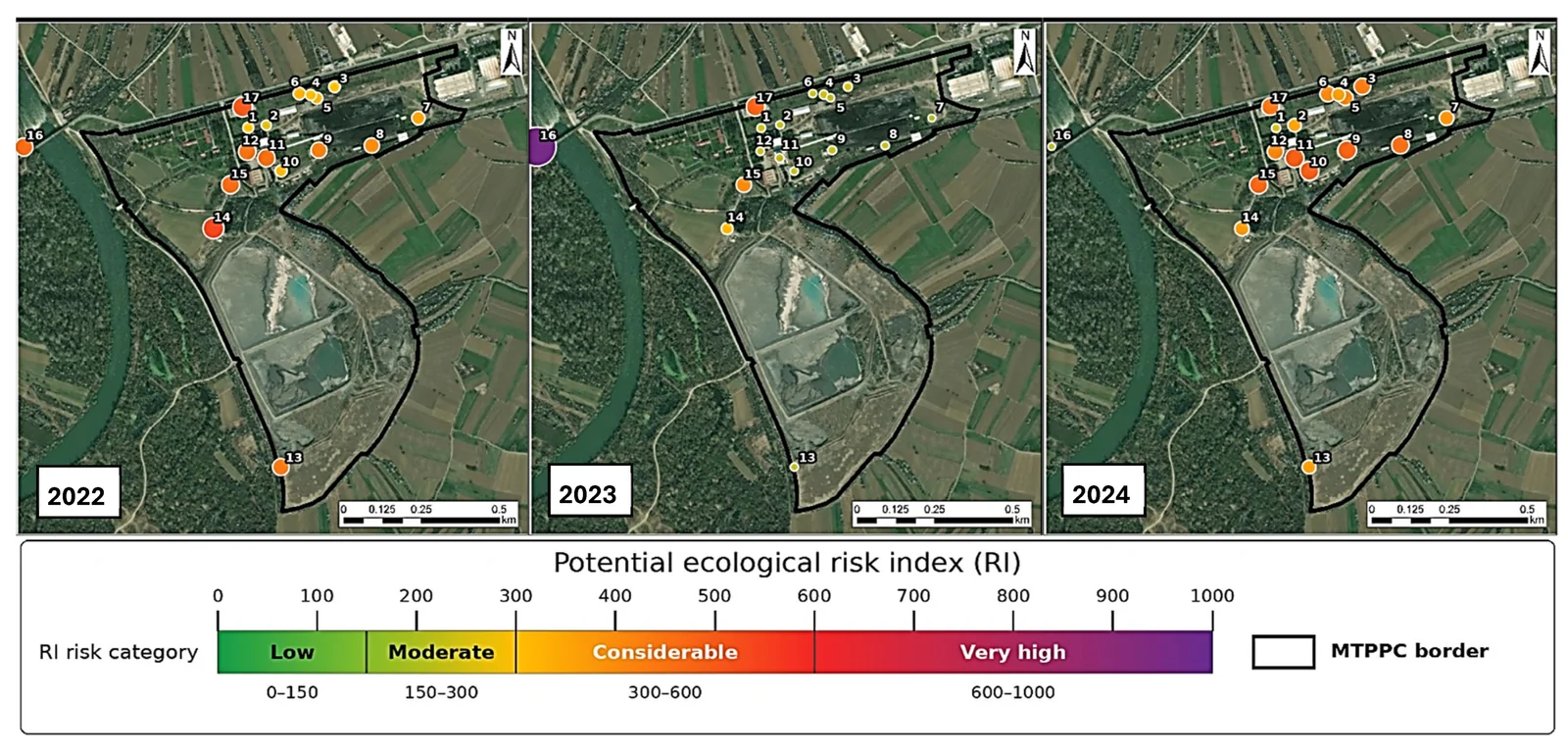
Potential ecological risk index (RI) values at the 17 predefined monitoring locations in 2022, 2023, and 2024.

The locations with the highest RI values also differed among the campaigns (Figure 5). In 2022, the highest RI was recorded at Z14, the pumping station at the ash landfill (535.14), followed by Z17, the parking area behind the switchyard (501.32), Z15, the plateau between the pumping station and the MTPPC fence (464.89), and Z16, below the bridge over the Great Morava River (462.29). In 2023, the very-high-risk value at Z16 (963.08) was substantially higher than all other values, followed by Z17 (478.93), Z15 (402.37), and Z14 (317.39). In 2024, the highest RI values shifted toward operational and process-related locations, including Z11 at the garage (487.92), Z10 behind the electrostatic precipitator facility (485.98), Z9 at the liquid fuel tanks (475.07), and Z15 (471.18). Comparatively elevated RI values were therefore recorded at ash-landfill-associated, operational and storage, electrical and process, and peripheral locations, rather than being confined to a single functional area.

Considerable ecological-risk values were recorded at multiple monitoring locations in each campaign. The element-specific potential ecological risk factors (Ei) showed that Cd and Hg were the principal contributors to the overall RI. Pb made a smaller secondary contribution, whereas the contributions of As, Cu, Cr, Ni, and Zn were generally lower (Figure 4).

### 3.4. Exploratory Associations Between Soil Properties and Total Metal and Metalloid Concentrations

Exploratory Spearman correlation analysis based on the 17 location-level median values showed several moderate-to-strong correlations based on unadjusted two-sided *p*-values (Table 9). The strongest negative correlations were observed between CaCO_3_ and Zn (ρ = −0.686, *p* = 0.002) and between CaCO_3_ and Pb (ρ = −0.659, *p* = 0.004). Pb was positively correlated with MWD (ρ = 0.637, *p* = 0.006), GMD (ρ = 0.608, *p* = 0.010), and clay content (ρ = 0.553, *p* = 0.021), and negatively correlated with pH measured in KCl (ρ = −0.540, *p* = 0.025). All other associations failed to meet the unadjusted significance threshold, including the positive coefficients of Cr and Ni with MWD and GMD (Table 9).

Additional positive correlations based on unadjusted *p*-values were observed between CEC and Cu (ρ = 0.596, *p* = 0.012) and between CaCO_3_ and Hg (ρ = 0.577, *p* = 0.015). However, none of the 56 evaluated correlations remained statistically significant after adjustment for multiple comparisons using the Benjamini–Hochberg procedure.

Accordingly, the correlations identified using unadjusted *p*-values were interpreted only as exploratory location-level patterns and not as confirmed controls on contaminant distribution. The relatively small number of monitoring locations, tied values associated with censored observations, and the predefined monitoring-network design further limit their interpretation.

### 3.5. Human Health Risk Based on HI and TCR

Across the 17 predefined monitoring locations, the aggregate Hazard Index (HI) ranged from 0.217 to 0.472 in 2022, from 0.211 to 0.476 in 2023, and from 0.247 to 0.484 in 2024. All HI values remained below the screening threshold of 1. Total carcinogenic risk (TCR) ranged from 5.98 × 10^−5^ to 1.56 × 10^−4^ in 2022, from 7.14 × 10^−5^ to 2.12 × 10^−4^ in 2023, and from 8.41 × 10^−5^ to 1.66 × 10^−4^ in 2024. All TCR values exceeded the target risk benchmark of 1 × 10^−6^, by approximately 60–212 times.

**Figure 6:**
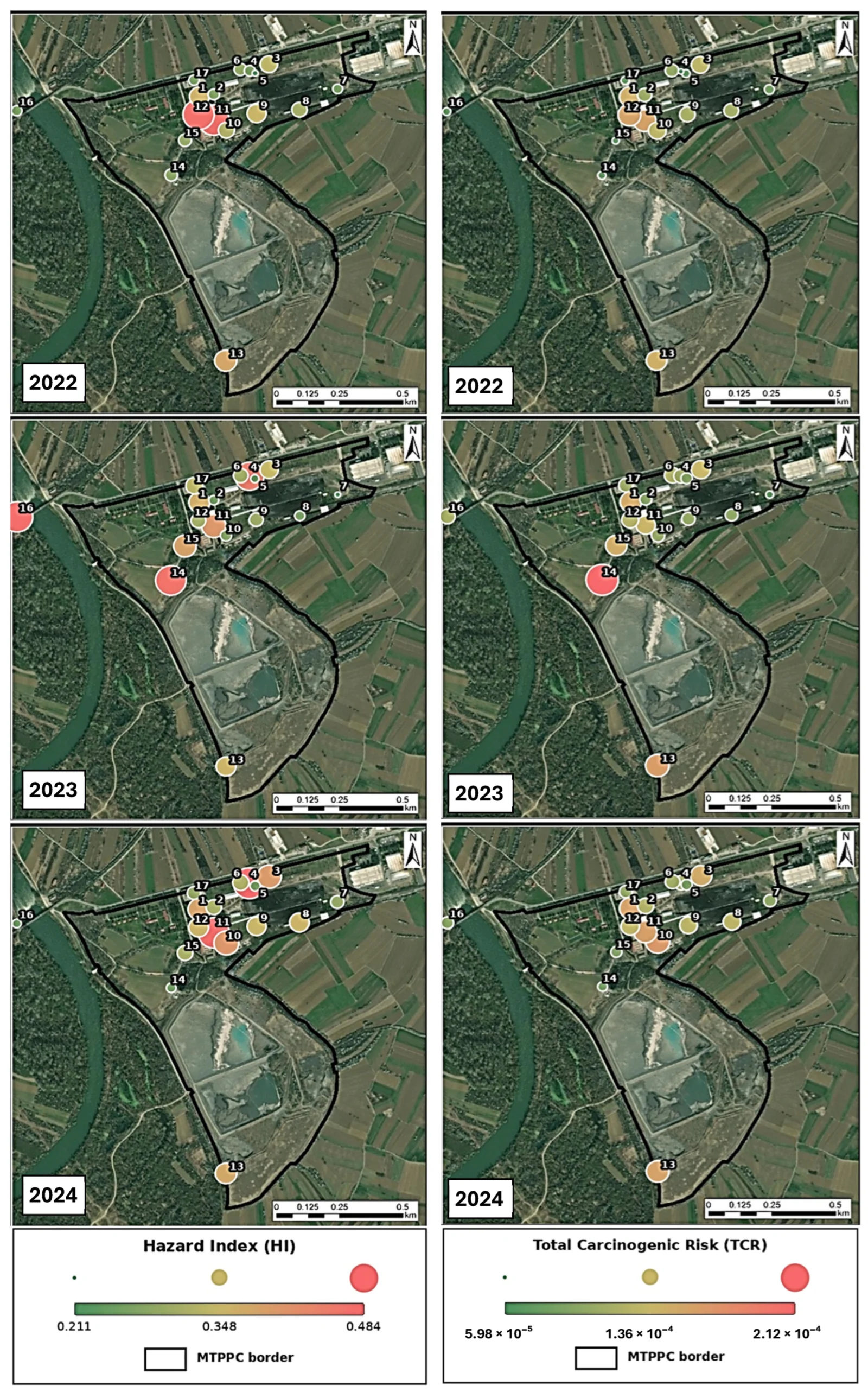
HI and TCR values for the Outdoor Worker scenario at the 17 predefined monitoring locations in 2022–2024. Screening benchmarks were HI = 1 and TCR = 1 × 10^−6^.

The locations with the highest HI values differed among the monitoring campaigns (Figure 6). In 2022, the maximum HI of 0.472 was recorded at both Z11, located at the garage, and Z12, located in the green area in front of the water chemical treatment plant. In 2023, the highest HI was recorded at Z16 below the bridge over the Great Morava River (0.476), followed by Z14 at the ash-landfill pumping station (0.466) and Z4 behind the technical gas storage area (0.453). In 2024, the highest values occurred at Z4 (0.484) and Z11 (0.479). Thus, comparatively elevated non-carcinogenic risk estimates were not restricted to one monitoring location or functional area.

The highest TCR values also occurred in different parts of the monitored network (Figure 6). In 2022, the maximum TCR of 1.56 × 10^−4^ was recorded at Z11 and Z12. In 2023, the highest TCR was recorded at Z14 at the ash-landfill pumping station (2.12 × 10^−4^), followed by Z13 at ash-landfill Cell 5 (1.62 × 10^−4^). In 2024, the maximum was recorded at Z10 behind the electrostatic precipitator facility (1.66 × 10^−4^), followed by Z11 (1.60 × 10^−4^), Z1 at the switchyard (1.57 × 10^−4^), and Z13 (1.56 × 10^−4^). Comparatively elevated TCR values were therefore recorded within electrical and process infrastructure, storage and operational areas, and ash-landfill-associated locations, rather than being confined to a single part of the complex.

**Figure 7:**
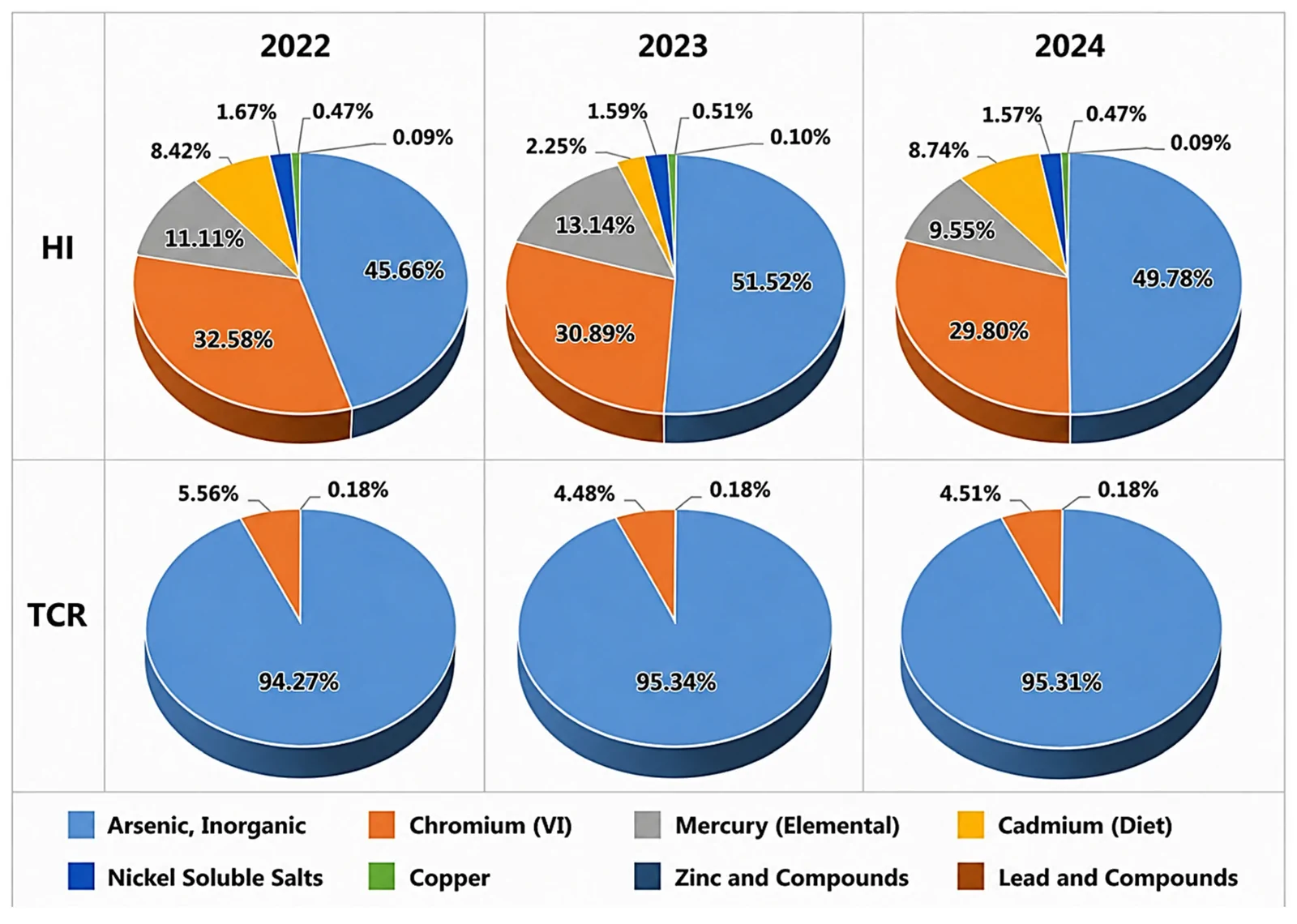
Campaign-level relative contributions of individual contaminants to the Hazard Index (HI) and total carcinogenic risk (TCR) for the Outdoor Worker exposure scenario during the 2022, 2023, and 2024 monitoring campaigns. Contributions were calculated from the contaminant-specific risk estimates and expressed as percentages of the corresponding aggregate HI or TCR. Percentages may not sum to exactly 100% due to rounding. A common color scheme and legend were retained across all panels to ensure consistency; consequently, some categories listed in the legend are not present in every individual chart.

The campaign-level relative composition of HI was dominated by As, evaluated using the RAIS toxicity parameters for inorganic arsenic, and by the conservative Cr(VI)-based estimate (Figure 7). As accounted for 45.66% of HI in 2022, 51.52% in 2023, and 49.78% in 2024, while the corresponding Cr(VI)-based contributions were 32.58%, 30.89%, and 29.80%. Hg contributed 11.11%, 13.14%, and 9.55%, respectively, while Cd contributed 8.42%, 2.25%, and 8.74%. The combined contributions of the remaining evaluated contaminants were approximately 2% in each campaign.

The composition of TCR was more strongly concentrated. As accounted for 94.27% of TCR in 2022, 95.34% in 2023, and 95.31% in 2024. The conservative Cr(VI)-based estimate was the principal secondary contributor, accounting for 5.56%, 4.48%, and 4.51%, respectively. Pb contributed approximately 0.18–0.19% in each campaign, while the contributions of the remaining contaminants were negligible (Figure 7).

**Figure 8:**
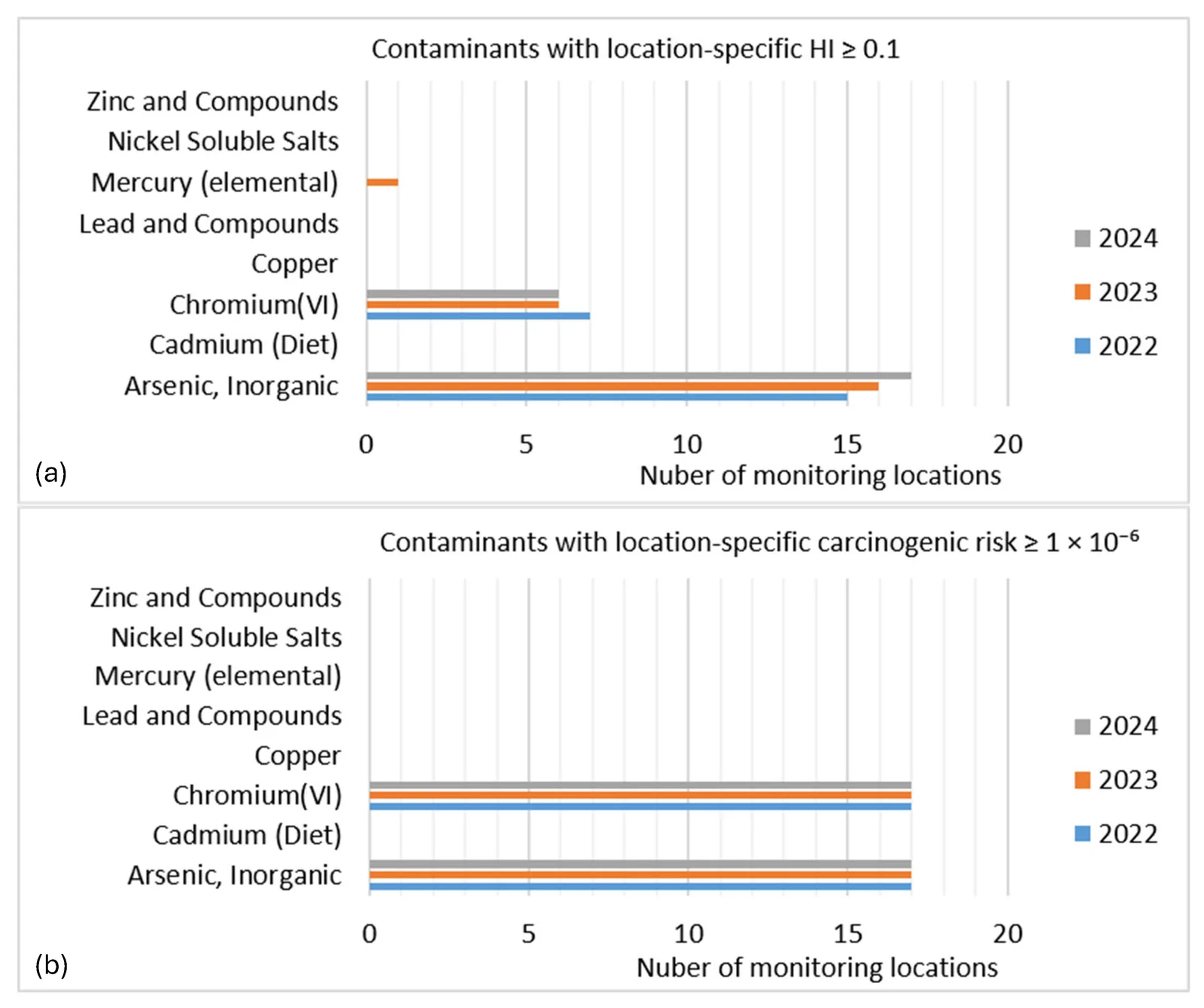
Number of predefined monitoring locations at which individual contaminants met the adopted screening-relevance criteria for (**a**) non-carcinogenic risk, defined as a contaminant-specific HI ≥ 0.1, and (**b**) carcinogenic risk, defined as a contaminant-specific risk ≥ 1 × 10^−6^, during the 2022, 2023, and 2024 monitoring campaigns.

Based on the adopted contaminant-specific non-carcinogenic risk criterion of a contribution to HI ≥ 0.1, As was identified as a screening-relevant contributor at 15 monitoring locations in 2022, 16 locations in 2023, and all 17 locations in 2024 (Figure 8a). The conservative Cr(VI)-based contribution met this criterion at seven locations in 2022 and at six locations in both 2023 and 2024. Hg met the criterion at one location in 2023 and at no locations in 2022 or 2024. None of the remaining contaminants met the adopted criterion.

For carcinogenic risk, As and the conservative Cr(VI)-based estimate were the only contaminant-specific contributions that reached or exceeded 1 × 10^−6^ (Figure 8b). Both met this criterion at all 17 monitoring locations in each of the three campaigns, while none of the remaining contaminants individually reached the adopted benchmark.

### 3.6. Integrated Comparison and Contaminant Prioritization Across Assessment Approaches

The integrated comparison showed that the three assessment approaches identified different contaminant priorities. Regulatory classification identified Ni and Cd as the most widespread concerns, whereas the potential ecological risk assessment was driven primarily by Cd and Hg. In the human health assessment, all HI values remained below 1, while all TCR estimates exceeded the adopted target benchmark; As was the dominant contributor, followed by the conservative Cr(VI)-based estimate.

These contrasting outcomes demonstrate that regulatory exceedance, toxicity-weighted ecological risk, and exposure-based human health risk represent complementary rather than interchangeable assessment endpoints. Consequently, contaminant prioritization within the MTPPC depends on the endpoint considered, supporting joint interpretation of the three assessment approaches rather than reliance on any single indicator.

## 4. Discussion

### 4.1. Location-Level Variability in Soil Properties and Contaminant Concentrations

The location-level variability observed in soil properties and contaminant concentrations within the MTPPC likely reflects the combined influence of heterogeneous alluvial parent material and long-term industrial land use (Table 6, Table 7 and Table 8). The study area is developed on stratified Quaternary deposits composed of variable proportions of gravel, sand, silt, and clay, which provide a natural basis for differences in soil texture, water-retention properties, porosity, and cation exchange capacity among the monitored locations [28,31]. At the same time, more than five decades of coal combustion, ash disposal, material storage, operational traffic, and surface disturbance may have modified local soil conditions and contributed additional contaminant inputs. However, because the monitoring locations were purposively selected and the functional groups were used only for descriptive interpretation, the observed differences cannot be attributed uniquely to particular operational activities or contamination sources.

The comparatively wide variation in clay content, water-retention properties, bulk density, penetration resistance, and aggregate-size indices indicates substantial differences in physical soil conditions among the monitoring locations (Table 6). Such differences may affect contaminant retention and redistribution because finer particles and soil aggregates provide surfaces for the sorption of metals, whereas porosity, compaction, and water movement influence infiltration, runoff, and the transport of particle-associated contaminants [4,5,18]. In the present study, MWD and GMD were used to summarize aggregate-size distribution and should not be interpreted as direct measures of aggregate stability or as evidence of progressive structural degradation. Their variation nevertheless indicates that the relative proportions of coarse and fine aggregates differed considerably among the monitored locations.

Soil chemical conditions also varied among locations, although pH was comparatively stable and generally ranged from near-neutral to moderately alkaline values (Table 7). In principle, alkaline conditions and the presence of carbonates may reduce the mobility of some cationic metals through adsorption, precipitation, and surface-complexation processes, whereas higher clay content and cation exchange capacity may increase their retention in the solid phase [3,4,9]. Nevertheless, these mechanisms were not measured directly in the present study and should not be inferred solely from total concentrations. In addition, all measurements of the operationally defined available fractions of Pb, Cd, Hg, As, Cr, Cu, Ni, and Zn were below the corresponding analytical detection limits. This finding indicates only that the extracted fractions were below the detection capability of the applied methods and does not demonstrate zero concentrations, complete immobilization, or negligible biological availability.

At the MTPPC, the relevant natural framework is the stratified alluvial parent material formed by successive fluvial deposition rather than a uniform soil-forming substrate. Coarser channel deposits dominated by gravel, sand, and silty sand and finer oxbow deposits composed of sandy-clayey and silty material may differ in their natural element contents, sorption capacity, and hydraulic behavior. Finer materials with higher clay content and CEC provide more surfaces for metal retention, while locally higher CaCO3 contents and near-neutral to moderately alkaline conditions may further reduce the mobility of some cationic metals through adsorption, surface complexation, or precipitation. Conversely, coarser or disturbed materials may permit greater infiltration and redistribution of dissolved or particle-associated contaminants [3,4,28].

Industrial disturbance is superimposed on this heterogeneous natural framework. Atmospheric deposition of fly ash particles, ash handling, material storage, operational traffic, and surface disturbance may introduce or redistribute metal-bearing particles in the surface soil, while historical ash disposal without impermeable liners or dedicated leachate control creates a potential pathway for downward transport. The resulting contaminant distribution may therefore reflect the interaction between the magnitude and location of industrial inputs and the contrasting retention or transport properties of the underlying alluvial materials. Fine-textured or more carbonate-rich locations may retain a larger fraction of particle-associated contaminants in surface soil, whereas coarser, more permeable, or disturbed zones may favor their redistribution. However, these pathways represent a site-based conceptual interpretation rather than demonstrated mechanisms because mineralogical composition, contaminant speciation, source fingerprints, leachate chemistry, and concurrent groundwater quality were not measured.

Total metal and metalloid concentrations differed among the predefined monitoring locations, with particularly pronounced relative variation observed for Cu, Hg, and Cr (Table 8). The highest location-level median Cu concentration was recorded at Z5 adjacent to the hydrogen storage area, whereas the maximum Cr concentration occurred at Z4 behind the technical gas storage area. These observations identify locations with comparatively elevated total concentrations but do not establish the origin of the contaminants. Similar studies conducted around Serbian coal-fired power plants have shown that contaminant occurrence may reflect a combination of atmospheric deposition, coal and ash handling, waste-management activities, local soil properties, and geogenic contributions from the parent material [6,8,23]. Distinguishing among these influences would require site-specific background values, source-material characterization, contaminant speciation, and formal source-apportionment analyses.

The exploratory correlation analysis provided limited evidence regarding the relationships between soil properties and total contaminant concentrations (Table 9). Based on unadjusted *p*-values, Pb was positively correlated with clay content, MWD, and GMD and negatively correlated with CaCO_3_ and pH measured in KCl, while additional correlations were observed between CEC and Cu, CaCO_3_ and Hg, and CaCO_3_ and Zn. These relationships may reflect differences in sorption conditions, parent material, or contaminant inputs among locations. However, none of the 56 evaluated correlations remained statistically significant after Benjamini–Hochberg adjustment. They should therefore be interpreted only as exploratory patterns and not as confirmed retention mechanisms or independent controls on contaminant distribution.

The relationships involving the aggregate-size indices merit additional consideration. In the present location-level analysis, only the positive relationships of Pb with MWD and GMD met the nominal unadjusted significance thresholds. The corresponding coefficients for Cr and Ni were also positive, but were weaker and not statistically significant. Moreover, none of these relationships remained significant after correction for multiple comparisons. The results therefore do not provide evidence that Cr, Pb, or Ni directly increased aggregate stability. Instead, the common positive direction of the coefficients may reflect shared variation in soil composition, contaminant retention, and aggregate-size distribution among the monitored locations.

Several non-exclusive processes could account for these patterns. Fine metal-bearing particles may be incorporated or physically occluded within larger dry aggregates, while metal concentrations and aggregate-size distribution may both vary in relation to clay content, parent-material mineralogy, organic binding agents, Fe and Mn oxides, carbonate content, compaction, and surface disturbance [43,44]. The nominal positive relationship between Pb and clay content supports the possibility that particle-associated Pb was retained by finer soil constituents subsequently incorporated into larger aggregates [43]. The weaker positive coefficients for Cr and Ni may reflect covariation with the texture or mineralogical composition of the heterogeneous alluvial parent material [23,28]. However, MWD and GMD were derived from dry-sieved aggregate fractions and describe aggregate-size distribution rather than resistance to breakdown under wet conditions [45]. In addition, metals were measured in bulk soil and not separately within individual aggregate fractions. Consequently, the present data cannot determine whether these elements were preferentially associated with particular aggregate sizes or whether they affected aggregate formation or stability. Confirmation would require wet-sieving measurements of water-stable aggregates, fraction-specific metal analyses, and supporting measurements of organic carbon, oxide mineralogy, and contaminant speciation.

### 4.2. Ecological Risk (RI) and Contaminant Prioritization

The regulatory classification identified Ni and Cd as the contaminants with the most widespread exceedance patterns within the monitored network (Figure 3). Ni exceeded the applicable limit value at all 17 monitoring locations in each campaign, with remediation values exceeded at three locations in 2022 and one location in 2024. Cd also exceeded the limit value at most locations, although no Cd concentration exceeded the remediation value. Exceedances of Pb, Cr, Cu, and Zn were recorded in all three campaigns but affected fewer locations, whereas As and Hg exceedances were more limited. PAHs remained below the applicable limit values throughout the monitoring period, and mineral oil fractions exceeded the limit value at only one location in 2022. These results indicate that regulatory non-compliance was primarily associated with metals rather than with the evaluated organic pollutants.

The regional studies also demonstrate that the identification of key contaminants depends on the investigated setting and assessment endpoint. Ćujić et al. (2016) [8] identified Ni as the element with the highest contamination factor around the Nikola Tesla power plants, followed by Zn, Co, and Cd. In agricultural alluvial soils around the Kostolac power plant, Šajnović et al. [23] attributed most As, Cu, and Pb concentrations to anthropogenic influences, whereas Cd, Co, Cr, Ni, and Zn were interpreted predominantly in relation to geological sources. Using a resident-exposure scenario around Nikola Tesla A, Tanić et al. [24] highlighted Co, Fe, and Mn in relation to non-carcinogenic risk for children and Cr and Pb as important contributors to carcinogenic risk. In the present study, the regulatory assessment prioritized Ni and Cd, the toxicity-weighted RI was driven mainly by Cd and Hg, and As accounted for more than 94% of TCR under the Outdoor Worker scenario. These differences illustrate the specific contribution of the integrated framework: the contaminant identified as most important depends on whether regulatory exceedance, toxicity-weighted ecological risk, or scenario-specific human exposure is evaluated.

The widespread Ni exceedances require cautious interpretation. Although coal combustion, atmospheric deposition, ash handling, fuel storage, and other industrial activities may contribute to metal inputs, total Ni concentrations may also reflect the mineralogical and geochemical characteristics of the alluvial parent material. Previous investigations around coal-fired power plants in Serbia have similarly reported elevated Ni and other trace-element concentrations and have emphasized the difficulty of separating industrial contributions from regional geogenic backgrounds using concentration data alone [6,8,23]. The present monitoring design therefore identifies locations and contaminants requiring attention but does not provide a basis for assigning individual exceedances to specific sources.

The potential ecological risk assessment produced a different contaminant prioritization from the regulatory classification because RI incorporates both concentration relative to a selected background value and element-specific toxic-response factors. Moderate and considerable RI categories were recorded during all three campaigns, while the only very-high-risk value occurred at Z16 in 2023 (Figure 4 and Figure 5). The highest RI values were recorded in different parts of the monitored network: landfill-associated and peripheral locations were prominent in 2022 and 2023, whereas several operational, storage, and process-related locations had comparatively elevated values in 2024. The distribution therefore did not identify a single functional area as the exclusive focus of potential ecological risk.

Although Ni was the most widespread regulatory exceedance, it made a comparatively limited contribution to RI because its toxic-response factor was lower than those assigned to Cd and Hg. In contrast, Cd and Hg were the principal contributors to the overall RI despite their different regulatory exceedance patterns (Figure 4). This contrast illustrates an important distinction between the two approaches. Regulatory classification identifies concentrations that exceed legally prescribed values, whereas RI additionally weights the degree of enrichment by the assumed relative toxicity of each element. Consequently, a contaminant may be prominent in the regulatory assessment without dominating RI, while another contaminant may contribute strongly to RI even when its regulatory exceedances are less widespread.

The dominant contribution of Cd and Hg to RI should nevertheless be interpreted in relation to the methodological assumptions of the index. The RI estimates depend on the selected reference background concentrations and toxic-response factors (Table 2), and the adopted background values may not fully represent the local geochemical baseline of the MTPPC area. In addition, RI is based on total element concentrations rather than chemical speciation, operationally defined availability, or directly measured biological effects. The influence of Hg is additionally sensitive to the treatment of values below the analytical detection limit because the full detection limit was conservatively used in the risk calculations. Consequently, the absolute RI values should be interpreted primarily as screening-level indicators for contaminant prioritization rather than as site-specific measures of ecological damage.

The operational indicators showed substantial overall reductions in electricity generation, coal and fuel-oil consumption, and reported atmospheric emissions between 2022 and 2024 (Table A5). However, widespread regulatory exceedances and moderate or considerable RI values were recorded throughout the same period. The annual operational data were not matched to the soil-sampling dates or individual monitoring locations and therefore cannot be used to establish a direct relationship between annual plant activity and soil-contaminant concentrations. The campaign-specific frequency of Cd limit-value exceedances (17, 11, and 16 monitoring locations in 2022, 2023, and 2024, respectively) did not parallel the marked decline in plant-wide operational indicators.

The lack of correspondence between the declining plant-wide operational indicators and the non-monotonic Cd exceedance frequency indicates only that the observed regulatory pattern cannot be explained by same-year operating intensity alone; it does not exclude historical or ongoing industrial contributions. Soil Cd may reflect cumulative deposition, legacy ash handling, redistribution of previously deposited material, local surface disturbance, and variation in soil retention and transport conditions. However, campaign-specific information on coal provenance and composition, changes in ash-disposal practices, and meteorological conditions matched to the soil-sampling dates was not available. Moreover, the operational dataset comprised only three plant-level annual observations. Consequently, these factors could not be evaluated as explanatory variables, and no statistical or causal relationship between plant operation and inter-campaign Cd variation was inferred.

Overall, the regulatory and ecological-risk assessments provide complementary information. The regulatory results identify Ni and Cd as the most widespread concerns relative to national soil-quality criteria, whereas the RI assessment prioritizes Cd and Hg because of their concentration-to-background ratios and high toxic-response factors. Joint interpretation is therefore necessary to distinguish regulatory non-compliance from toxicity-weighted potential ecological risk and to identify contaminants and monitoring locations requiring more detailed site investigation.

### 4.3. Human Health Implications

The human health risk assessment showed a clear distinction between non-carcinogenic and carcinogenic screening outcomes for the Outdoor Worker scenario. Aggregate HI values ranged from 0.211 to 0.484 and remained below the screening threshold of 1 at all monitoring locations and during all three campaigns (Figure 6). These results indicate that the combined non-carcinogenic exposure estimate did not exceed the adopted screening threshold under the applied exposure assumptions. In contrast, TCR ranged from 5.98 × 10^−5^ to 2.12 × 10^−4^ and exceeded the target benchmark of 1 × 10^−6^ at every monitoring location in each campaign (Figure 6). This contrast demonstrates that a result below the non-carcinogenic threshold does not imply an absence of concern for carcinogenic endpoints because HI and TCR evaluate different toxicological effects and are based on different toxicity parameters.

Comparatively elevated HI and TCR values were recorded in several functional parts of the monitored network rather than being restricted to the ash landfill. Higher estimates occurred at storage and operational locations, including Z4 and Z11; at electrical and process infrastructure, including Z1 and Z10; at ash-landfill-associated locations Z13 and Z14; and at green or peripheral locations, including Z12 and Z16 (Figure 6). The locations with the highest values also differed among campaigns. This distribution suggests that the calculated human health risk estimates cannot be attributed to a single operational sector or contamination source. The observed pattern may reflect differences in total contaminant concentrations, historical inputs, local material handling, surface disturbance, and soil characteristics, but the monitoring design does not permit formal source attribution.

The contaminant-specific contributions further illustrate the difference between concentration-based regulatory assessment and toxicological risk assessment. As accounted for approximately 46–52% of aggregate HI and more than 94% of TCR during the three campaigns, although As exceeded its regulatory limit value at relatively few monitoring locations (Figure 3 and Figure 7). By comparison, Ni was the most widespread regulatory exceedance but made only a limited contribution to the calculated human health risk. These differences arise because health risk estimates incorporate not only measured concentrations but also exposure assumptions and contaminant-specific toxicity values. Consequently, contaminants present at comparatively moderate concentrations may dominate risk estimates when their toxicological potency is high.

The conservative Cr(VI)-based estimate was the second most important contributor to both HI and TCR. It contributed approximately 30–33% of HI and 4.5–5.6% of TCR and met the contaminant-specific screening criteria at several or all monitoring locations, depending on the evaluated endpoint (Figure 7 and Figure 8). However, chromium speciation was not determined analytically, and the measured total Cr concentrations were evaluated using Cr(VI) toxicity parameters. The resulting Cr-related estimates should therefore be interpreted as conservative upper-bound screening values and not as evidence that the measured chromium occurred entirely, or predominantly, as Cr(VI). Direct determination of Cr species would be required to refine the Cr-related component of the assessment.

The dominance of As and the conservative Cr(VI)-based estimate also explains why the contaminants prioritized by the human health assessment differed from those prioritized by RI. Cd and Hg dominated the potential ecological risk index because of their concentration-to-background ratios and high toxic-response factors, whereas As and the Cr(VI)-based estimate dominated the human health calculations because of the toxicity values and exposure assumptions applied in RAIS (Figure 4 and Figure 7). These results confirm that regulatory classification, ecological-risk assessment, and human health risk assessment describe different aspects of contamination and should not be treated as interchangeable indicators.

The calculated HI and TCR values represent screening-level estimates rather than measured health effects or predictions of disease occurrence. The assessment was limited to the Outdoor Worker scenario and to exposure through incidental soil ingestion, dermal contact, and inhalation of resuspended soil particles. It did not evaluate residential or child receptors, dietary intake, groundwater or surface-water exposure, plant uptake, contaminant bioavailability, or actual individual exposure. The exceedance of the TCR benchmark therefore indicates the need for more detailed site-specific evaluation, particularly of As and chromium speciation, rather than demonstrating that adverse health effects have occurred. Further assessment should include refined exposure parameters, contaminant speciation and bioavailability, and, where relevant, additional environmental media and receptor scenarios.

### 4.4. Implications for Ecological Monitoring and Reclamation Planning

The contrasting priorities identified by the regulatory, ecological-risk, and human health assessments demonstrate the value of combining several complementary approaches within the MTPPC monitoring program (Table 5). Regulatory classification provided a direct indication of compliance with national soil-quality criteria, RI incorporated concentration relative to selected background values and element-specific toxic-response factors, and HI and TCR evaluated potential exposure under the Outdoor Worker scenario. Because these approaches address different endpoints and rely on different assumptions, none of them alone provides a complete characterization of environmental condition or risk. Their joint interpretation offers a more informative basis for identifying contaminants and monitoring locations that require further investigation.

The present results indicate that future monitoring should continue to include both soil properties and contaminant concentrations. Variation in texture, pH, carbonate content, cation exchange capacity, porosity, compaction, water-retention properties, and aggregate-size distribution may influence contaminant retention and redistribution. Monitoring programs based only on total contaminant concentrations may therefore overlook site conditions that affect contaminant mobility and potential exposure. However, the exploratory correlations obtained in this study did not remain statistically significant after correction for multiple comparisons (Table 9), and soil properties should consequently be treated as supporting interpretative variables rather than as confirmed controls on contaminant distribution.

The predefined monitoring network provided repeated information from the same 17 locations but did not support spatial interpolation or identification of spatially continuous contamination zones. Comparatively elevated contaminant concentrations and risk estimates occurred in several functional parts of the complex, including electrical and process infrastructure, storage and operational areas, ash-landfill-associated locations, and peripheral locations. Future monitoring should therefore retain the existing locations to preserve comparability while considering additional targeted sampling around locations with remediation-value exceedances, comparatively elevated RI, HI, or TCR values, and areas affected by ash handling, drainage, storage, or intensive surface disturbance. Additional sampling should be designed to define the local extent of these conditions rather than assuming that values recorded at individual monitoring locations represent the surrounding area.

The ecological-risk assessment applied in this study was based on total element concentrations, selected background concentrations, and toxic-response factors and did not directly measure biological effects. Consequently, RI should be interpreted as a screening-level estimate of potential ecological risk. Effect-based biotests could complement chemical analyses by providing information on the combined biological effects of contaminant mixtures and the fraction of contamination that is biologically accessible under the test conditions. Assays based on luminescent bacteria, together with plant, invertebrate, or other ecotoxicological tests, may be particularly useful where chemical concentrations and toxicity-weighted indices provide different contaminant priorities. Such tests would not replace chemical monitoring, but could help determine whether locations with elevated RI values also produce measurable biological responses.

The planned reclamation of the ash landfill provides an important practical context for continued monitoring. The present three-campaign dataset can serve as a pre-reclamation reference for the monitored locations, but it should not be interpreted as a complete characterization of the landfill or the wider complex. Monitoring before, during, and after reclamation should use consistent sampling locations, depths, analytical methods, and detection limits wherever possible. Additional measurements should include vegetation establishment, soil physical condition, contaminant concentrations, and appropriate ecological-risk or effect-based indicators. This would allow future changes to be evaluated against the present pre-reclamation reference and help distinguish improvements associated with reclamation from differences caused by analytical methods or short-term environmental variability.

The hydrogeological setting of the MTPPC represents a vertically heterogeneous system with different implications for contaminant retention and transport. The approximately 4–6 m thick cover of sandy and clayey silts has reported hydraulic-conductivity values ranging from 10^−7^ to 10^−3^ cm s^−1^. The finer and less permeable portions of this cover may locally retard vertical percolation and favor the retention of particle-associated or sorbed contaminants. In contrast, sandier zones, disturbed material, or preferential flow paths may permit more rapid infiltration. The generally near-neutral to moderately alkaline soil reaction, together with variable carbonate content, clay content, and cation-exchange capacity, may additionally favor the retention of some cationic metals through adsorption, surface complexation, or precipitation, although these processes were not measured directly and cannot be inferred from total concentrations alone [3,4,9].

If dissolved or colloid-associated contaminants pass through the upper fine-grained cover, the underlying 2–5 m thick gravelly sandy aquifer, with reported hydraulic-conductivity values of approximately 10^−3^ to 10^−1^ cm s^−1^, would provide substantially greater potential for advective transport. The relatively impermeable Pliocene clay substratum may restrict deeper vertical migration and could favor lateral transport within the overlying permeable unit. The absence of impermeable liners and dedicated leachate-control systems during much of the historical operation of the ash landfill increases the relevance of these potential pathways. However, the described implications represent a conceptual interpretation of the documented stratigraphy and hydraulic properties and do not demonstrate that contaminants detected in surface soil have migrated into groundwater. Overall, the monitoring program should evolve from concentration-based soil surveillance toward a tiered framework combining regulatory assessment, toxicity-weighted screening, human health risk assessment, supporting soil-property measurements, and targeted effect-based testing. The current results provide a screening-level basis for selecting priorities, whereas confirmation of ecological effects, contaminant mobility, and the effectiveness of future reclamation measures will require additional site-specific investigation.

### 4.5. Strengths and Limitations

A principal strength of the study is the repeated assessment of the same 17 predefined locations across three annual campaigns using a broad analytical program and three complementary assessment frameworks. The principal methodological value lies in applying national regulatory classification, the potential ecological risk index, and RAIS-based Outdoor Worker scenario risk assessment to the same dataset, thereby allowing direct comparison of endpoint-specific contaminant priorities. The integration of contaminant measurements with soil physical, chemical, water-retention, mechanical, and aggregate-size properties provides a broader basis for interpretation than concentration-only assessments. The dataset also establishes a pre-reclamation reference for future monitoring and evaluation of reclamation measures at the MTPPC.

The monitoring network was purposively selected and did not include a probabilistically defined spatial grid or an independently established local geochemical baseline. Moreover, one composite surface-soil sample (0–30 cm) was analyzed per location and campaign. The dataset therefore cannot quantify within-location variability, vertical concentration profiles, or spatially continuous conditions, or formally distinguish geogenic from industrial contributions. The RI, HI, and TCR results are screening estimates based primarily on total concentrations and adopted background, exposure, and toxicity parameters; contaminant speciation, site-specific bioavailability, and biological effects were not determined. The adopted background concentrations may not fully represent the local geochemical baseline. The health-risk assessment was limited to the Outdoor Worker scenario and soil ingestion, dermal contact, and inhalation of resuspended particles; residential and child receptors, dietary intake, plant uptake, and actual individual exposure were not assessed. In addition, total Cr was conservatively evaluated using Cr(VI) toxicity parameters, which may overestimate the Cr-related risk. Finally, the correlation analysis was exploratory, and none of the evaluated associations remained significant after correction for multiple comparisons. Because MWD and GMD were calculated from dry-sieved fractions, they characterize aggregate-size distribution rather than resistance to breakdown under wet conditions. Metals were analyzed in bulk soil rather than in individual aggregate fractions, preventing conclusions about fraction-specific metal retention or direct effects on soil aggregation.

Mechanistic interpretation and source attribution were also limited by the absence of mineralogical data, Fe and Mn oxide measurements, contaminant speciation, source fingerprints, and chemical analyses of coal, fly ash, and leachate. Operational indicators were available only as three plant-level annual totals and were not matched to individual sampling dates or locations. Campaign-specific information on coal composition, ash-disposal practices, and meteorological conditions was also unavailable. Consequently, these data provided operational context but could not explain the inter-campaign Cd variation or establish causal industrial contributions.

The absence of groundwater-level and groundwater-quality measurements concurrent with the 2022–2024 soil-monitoring campaigns is a material limitation of the present assessment. Without campaign-specific groundwater elevations, hydraulic gradients, and chemical analyses, it was not possible to determine the vertical separation between the sampled surface soil and the groundwater table, establish groundwater-flow direction, evaluate seasonal groundwater–river interactions, or determine whether contaminants detected in soil had reached the underlying aquifer. Accordingly, the ecological and human health risk assessments presented here are soil-specific screening assessments. The evaluated Outdoor Worker scenario includes incidental soil ingestion, dermal contact, and inhalation of resuspended soil particles, but does not include groundwater ingestion or other water-related exposure pathways. The results should therefore not be interpreted as a complete multimedia risk assessment of the MTPPC. Future monitoring should combine soil sampling with seasonal groundwater-level measurements and chemical analyses of groundwater and drainage water, preferably at hydraulically upgradient and downgradient locations.

The three annual monitoring campaigns do not support a formal temporal-trend analysis. A substantially longer and methodologically consistent monitoring record, ideally comprising at least 10 annual campaigns, would provide a stronger basis for determining whether the location-specific observations and contaminant priorities persist over time.

## 5. Conclusions

This three-year case study integrated soil-quality data, regulatory classification, potential ecological risk, and outdoor worker human health risk assessment for 17 predefined monitoring locations within and immediately surrounding the MTPPC. The results revealed substantial location-level variation in soil physical and chemical properties and in total metal and metalloid concentrations. However, the monitoring design did not support formal temporal-trend analysis, spatially continuous estimation, or attribution of the observed contaminant patterns to individual industrial or natural sources.

Regulatory classification identified Ni and Cd as the most widespread concerns. Ni exceeded the applicable limit value at all 17 monitoring locations in each campaign, including several remediation-value exceedances, while Cd exceeded the limit value at most locations. Exceedances of Pb, Cr, Cu, and Zn were also recorded but affected fewer locations. The potential ecological risk assessment provided a different prioritization: moderate and considerable RI categories occurred in all three campaigns, and one very-high-risk value was recorded at Z16 in 2023. Cd and Hg were the principal contributors to RI, primarily because of their concentration-to-background ratios and high toxic-response factors.

The human health risk assessment distinguished clearly between non-carcinogenic and carcinogenic screening outcomes. Aggregate HI values ranged from 0.211 to 0.484 and remained below the screening threshold of 1 at all monitoring locations. In contrast, TCR ranged from 5.98 × 10^−5^ to 2.12 × 10^−4^ and exceeded the target benchmark of 1 × 10^−6^ at every location in all three campaigns. As was the dominant contributor to both endpoints and accounted for more than 94% of TCR. The Cr-related component, calculated conservatively by applying Cr(VI) toxicity parameters to measured total Cr concentrations, was the principal secondary contributor and should be refined through direct chromium-speciation analysis.

The different assessment approaches therefore provided complementary rather than interchangeable information. Ni and Cd were most prominent in relation to regulatory exceedances, Cd and Hg dominated the potential ecological risk index, and As and the conservative Cr(VI)-based estimate dominated the human health risk calculations. In this case study, contaminant prioritization depended on the evaluated endpoint, indicating that the assessment should not rely on concentration data or any single risk indicator alone.

The three-campaign dataset provides a screening-level pre-reclamation reference for continued environmental monitoring and for evaluating future reclamation activities at the MTPPC. Further investigation should retain the existing monitoring locations while adding targeted sampling around locations with remediation-value exceedances or comparatively elevated risk estimates. Priority needs include contaminant speciation and bioavailability assessment, integrated soil–groundwater and drainage-water monitoring, and effect-based ecotoxicological testing. These additions would allow the screening results reported here to be verified and would provide a stronger basis for assessing contaminant mobility, biological effects, and the effectiveness of future reclamation and management measures.

## Figures and Tables

**Table 1:** Soil properties and contaminants analyzed during the 2022–2024 MTPPC monitoring program.

Parameter Group	Parameter
Soil chemical properties	pH (H_2_O)
	pH (KCl)
	Electrical conductivity (EC)
	CaCO_3_
	Cation exchange capacity (CEC)
	Total nitrogen
	Available phosphorus
	Available potassium
Ions and water-soluble components	Ammonium nitrogen (NH_4_–N)
	Water-soluble calcium (Ca^2+^)
	Water-soluble magnesium (Mg^2+^)
	Nitrate (NO_3_^−^)
	Nitrite (NO_2_^−^)
Total metals and metalloids	Chromium (Cr)
	Nickel (Ni)
	Lead (Pb)
	Copper (Cu)
	Zinc (Zn)
	Cadmium (Cd)
	Arsenic (As)
	Mercury (Hg)
	Boron (B)
Available forms of metals and metalloids	Chromium (Cr)
	Nickel (Ni)
	Lead (Pb)
	Copper (Cu)
	Zinc (Zn)
	Cadmium (Cd)
	Arsenic (As)
	Mercury (Hg)
Organic pollutants	Polycyclic aromatic hydrocarbons (PAHs)
	Mineral oil fractions (C_6_–C_40_)
Aggregate-size distribution	Aggregate fraction > 10 mm
	Aggregate fraction 10–5 mm
	Aggregate fraction 5–3 mm
	Aggregate fraction 3–2 mm
	Aggregate fraction 2–1 mm
	Aggregate fraction 1–0.5 mm
	Aggregate fraction 0.5–0.25 mm
	Aggregate fraction < 0.25 mm
Soil water-retention properties	Water retention at field capacity, FC (−33 kPa)
	Water retention at wilting point, WP (−1500 kPa)
	Plant-available and readily available water
Soil physical and mechanical properties	Clay (<0.002 mm)
	Particle density
	Bulk density
	Total porosity
	Penetration resistance

Note: Total and available concentrations of metals and metalloids were reported on an elemental basis. The applied analytical methods did not distinguish individual ionic species or oxidation states.

**Table 2:** Toxic-response factors and reference background concentrations used to calculate the potential ecological risk index (RI).

Element	T_i_ (Dimensionless)	C_ref,i_ (mg/kg)
Cd	30	0.5
Hg	40	0.1
As	10	15
Pb	5	20
Cu	5	25
Ni	5	20
Cr	2	70
Zn	1	70

**Table 3:** Classification criteria for the individual potential ecological risk factor (E_i_) [25].

E_i_ Value	Risk Level
E_i_ < 40	Low
40 ≤ E_i_ < 80	Moderate
80 ≤ E_i_ < 160	Considerable
160 ≤ E_i_ < 320	High
E_i_ ≥ 320	Very high

**Table 4:** Classification criteria for the overall potential ecological risk index (RI) [25,41].

RI Value	Risk Level
RI < 150	Low
150 ≤ RI < 300	Moderate
300 ≤ RI < 600	Considerable
RI ≥ 600	Very high

**Table 5:** Overview of the principal inputs, evaluated endpoints, strengths, and limitations of the monitoring and assessment approaches applied in this study.

Assessment Approach	Principal Inputs	Evaluated Endpoint	Main Strengths	Main Limitations
Repeated soil-monitoring design	Repeated soil sampling at the same 17 predefined monitoring locations, a consistent sampling depth, and a comparable analytical program across three annual campaigns	Location-specific patterns of soil contamination and risk indicators across the three monitoring campaigns	Enables repeated evaluation of the same monitoring locations and comparison of a broad set of soil properties and contaminants across campaigns	Limited to three annual campaigns and a predefined monitoring network; does not support formal long-term trend analysis, spatially continuous estimation, or causal source attribution
Regulatory threshold classification	Total concentrations of metals and metalloids, measured concentrations of selected organic pollutants, and prescribed limit and remediation values	Regulatory exceedance status at each monitoring location and campaign	Provides a direct and transparent comparison with national regulatory criteria	Evaluates contaminants individually and does not account for toxicity-weighted combined effects, exposure pathways, or site-specific bioavailability
Individual potential ecological risk factor (E_i_) and potential ecological risk index (RI)	Total element concentrations, reference background concentrations, and element-specific toxic-response factors	Element-specific and cumulative potential ecological risk	Integrates concentration relative to the selected background value with element-specific toxicity and identifies dominant contributors to RI	Screening-level approach dependent on the selected background concentrations and toxic-response factors; does not account for chemical speciation, bioavailability, mixture interactions, or directly measured biological effects
Hazard Index (HI)	Total concentrations of contaminants included in the RAIS assessment, scenario-specific exposure parameters, and non-carcinogenic toxicity values	Aggregate non-carcinogenic risk for an outdoor worker	Incorporates multiple soil-exposure pathways and enables evaluation of contaminant-specific and aggregate contributions	Depends on exposure assumptions and toxicological reference values; the use of total Cr with Cr(VI) toxicity parameters provides a conservative estimate; does not represent observed health effects
Total carcinogenic risk (TCR)	Total concentrations of contaminants included in the RAIS assessment, scenario-specific exposure parameters, and carcinogenic toxicity values	Aggregate lifetime carcinogenic risk for an outdoor worker	Provides a quantitative screening estimate of carcinogenic risk across the evaluated soil-exposure pathways	Depends on exposure assumptions, contaminant speciation, and toxicity parameters; the conservative Cr(VI) assumption may overestimate the Cr-related contribution; represents estimated rather than observed health risk

Note: The principal methodological sources include the applicable Serbian soil-monitoring and regulatory framework for the monitoring design and regulatory classification [35,40], Håkanson (1980) [25] and Hu et al. (2013) [41] for the potential ecological risk assessment, and Oak Ridge National Laboratory (2024) [42] for the RAIS-based human health risk assessment. Effect-based ecotoxicological testing, discussed as a complementary monitoring approach in Section 4.4.

**Table 6:** Descriptive statistics of location-level median soil physical, water-retention, mechanical, and aggregate-size properties derived from the 2022–2024 monitoring campaigns.

Parameter	Unit	n	Mean	Median	SD	Min	Max	CV
Clay (<0.002 mm)	%	17	26.96	24.10	11.79	11.00	47.60	43.73
Particle density	g/cm^3^	17	2.70	2.70	0.03	2.64	2.74	1.10
Total porosity	%	17	51.76	51.85	5.18	37.24	57.54	10.00
Water retention at field capacity, FC (−33 kPa)	%	17	34.62	31.75	10.45	18.53	48.33	30.20
Water retention at wilting point, WP (−1500 kPa)	%	17	17.63	15.26	7.69	6.82	30.13	43.62
Plant-available and readily available water	%	17	17.07	16.36	4.70	6.72	24.46	27.53
Bulk density	g/cm^3^	17	1.30	1.30	0.16	1.12	1.73	12.00
Penetration resistance	MPa	17	0.14	0.12	0.07	0.04	0.24	46.70
Mean weight diameter (MWD)	mm	17	4.63	4.86	1.76	1.72	7.69	38.14
Geometric mean diameter (GMD)	mm	17	2.63	2.57	1.42	0.72	5.02	54.16
Moisture content at 105 °C	%	17	3.64	3.56	0.81	2.18	5.86	22.33

Note: For each parameter, one median value was calculated per predefined monitoring location from the available annual observations recorded in 2022, 2023, and 2024. Descriptive statistics were calculated from the resulting 17 location-level median values using unrounded data. For moisture content at 105 °C, location-level medians were based on the available 2023 and 2024 observations because this parameter was not determined in 2022. SD, standard deviation; CV, coefficient of variation; FC, field capacity; WP, wilting point; MWD, mean weight diameter; GMD, geometric mean diameter.

**Table 7:** Descriptive statistics of location-level median soil chemical properties and water-soluble components derived from the 2022–2024 monitoring campaigns.

Parameter	Unit	n	Mean	Median	SD	Min	Max	CV
pH (H_2_O)	dimensionless	17	7.65	7.69	0.33	6.75	8.13	4.37
pH (KCl)	dimensionless	17	7.09	7.30	0.45	5.82	7.67	6.29
Electrical conductivity (EC)	mS/cm	17	0.43	0.37	0.30	0.23	1.52	69.36
CaCO_3_	%	17	1.62	1.41	1.40	0.19	4.91	86.76
Cation exchange capacity (CEC)	cmol(+)/kg	17	21.23	16.66	15.27	10.18	77.00	71.91
Total nitrogen	%	17	0.18	0.16	0.10	0.05	0.43	53.92
Available phosphorus	mg P_2_O_5_/100 g	17	5.44	0.81	8.31	0.50	32.80	152.66
Available potassium	mg K_2_O/100 g	17	31.49	32.41	13.78	9.60	55.18	43.75
Ammonium nitrogen (NH_4_–N)	mg/kg	17	7.12	6.98	4.08	0.25	15.70	57.36
Water-soluble calcium (Ca^2+^)	mg/kg	17	131.45	89.70	167.90	38.10	771.50	127.73
Water-soluble magnesium (Mg^2+^)	mg/kg	17	22.66	20.14	11.23	10.34	56.92	49.58
Nitrate (NO_3−_)	mg/kg	17	4.29	4.30	2.79	0.50	9.45	64.91

Note: For each parameter, one median value was calculated per predefined monitoring location from the available annual observations recorded in 2022, 2023, and 2024. Descriptive statistics were calculated from the resulting 17 location-level median values using unrounded data. For descriptive aggregation, annual observations reported below the limit of detection were replaced by one-half of the corresponding detection limit (LOD/2) before calculating location-level medians for available phosphorus, total nitrogen, ammonium nitrogen, and nitrate. Nitrite was excluded from the quantitative location-level summary because observations below the limit of detection predominated. SD, standard deviation; CV, coefficient of variation; EC, electrical conductivity; CEC, cation exchange capacity.

**Table 8:** Descriptive statistics of location-level median total concentrations of selected metals and metalloids derived from the 2022–2024 monitoring campaigns.

Parameter	Unit	n	Mean	Median	SD	Min	Max	CV (%)
Cadmium (Cd)	mg/kg	17	2.69	2.80	0.88	0.40	3.50	32.66
Mercury (Hg)	mg/kg	17	0.29	0.27	0.16	0.13	0.60	57.29
Arsenic (As)	mg/kg	17	17.18	16.65	3.54	12.54	23.55	20.59
Copper (Cu)	mg/kg	17	39.22	34.20	23.42	19.82	120.18	59.72
Lead (Pb)	mg/kg	17	87.51	87.60	25.33	49.60	137.41	28.95
Chromium (Cr)	mg/kg	17	122.59	106.30	60.15	74.72	321.47	49.06
Nickel (Ni)	mg/kg	17	137.32	135.50	26.69	95.34	174.14	19.43
Zinc (Zn)	mg/kg	17	114.34	118.63	23.60	72.60	142.41	20.64

Note: For each element, one median value was calculated per predefined monitoring location from the available annual observations recorded in 2022, 2023, and 2024. Descriptive statistics were calculated from the resulting 17 location-level median values using unrounded data. Annual concentrations reported below the limit of detection were replaced by one-half of the corresponding detection limit (LOD/2) before calculating the location-level medians. For Cd and Hg, values reported as <0.8 and <0.25 mg/kg were represented by 0.40 and 0.125 mg/kg, respectively. SD, standard deviation; CV, coefficient of variation. Concentrations are reported on an elemental basis. The applied analytical methods did not distinguish individual ionic species or oxidation states.

**Table 9:** Spearman rank correlation coefficients between selected soil properties and total concentrations of selected metals and metalloids, based on 17 location-level median values.

Soil Property	Cd	Hg	As	Cu	Pb	Cr	Ni	Zn
Clay (%)	−0.041	0.161	0.102	−0.136	0.553 *	0.219	0.343	0.174
CEC (cmol(+)/kg)	−0.476	−0.451	0.416	0.596 *	0.215	0.413	0.278	0.124
pH (H_2_O)	−0.085	0.416	−0.270	−0.249	−0.436	−0.294	−0.304	−0.270
pH (KCl)	−0.163	0.129	−0.179	0.102	−0.540 *	−0.287	−0.426	−0.306
CaCO_3_ (%)	0.126	0.577 *	−0.328	−0.428	−0.659 **	−0.382	−0.439	−0.686 **
MWD (mm)	0.003	0.028	0.203	−0.059	0.637 **	0.346	0.453	0.260
GMD (mm)	0.013	0.085	0.132	−0.104	0.608 **	0.255	0.360	0.230

Note: Spearman rank correlation coefficients were calculated from 17 location-level median values derived from the 2022–2024 monitoring campaigns. Asterisks identify associations meeting nominal significance thresholds before correction for multiple testing (* *p* < 0.05; ** *p* < 0.01) and are shown for exploratory purposes only. None of the 56 evaluated associations remained statistically significant after Benjamini–Hochberg false-discovery-rate adjustment (adjusted *p* < 0.05). Accordingly, these relationships should be interpreted as hypothesis-generating location-level patterns rather than confirmed associations. Blue shading indicates negative correlations and red shading indicates positive correlations; darker shades represent larger absolute correlation coefficients.

## Data Availability

The data supporting the findings of this study are available from the corresponding author upon reasonable request, subject to the applicable restrictions associated with the original environmental monitoring documentation.

## Appendix A

**Table A1:** Locations within the MTPPC where soil samples were collected.

Sampling Location Code	Description of Sampling Location
Z1	Switchyard, in front of the fence of transformer T5
Z2	Switchyard, in front of the fence of transformer T4 near the collection sump
Z3	Hazardous waste storage area
Z4	Plateau behind the technical gas storage area
Z5	Adjacent to the hydrogen storage area
Z6	Oil and chemical storage area
Z7	Plateau between the weighbridge and the unloading station at the coal landfill
Z8	Green area between the locomotive depot and the fence
Z9	Liquid fuel tanks
Z10	Plateau behind the electrostatic precipitator facility
Z11	Garage
Z12	Green area in front of the water chemical treatment plant
Z13	Ash landfill, cell No. 5
Z14	Pumping station at the ash landfill
Z15	Plateau between the pumping station and the MTPPC fence
Z16	Below the bridge over the Great Morava River
Z17	Parking area behind the switchyard

**Table A2:** Methods for the analysis of soil properties for 2022.

Parameter Group	Analyzed Parameter	Regulation, Method or Standard
Soil chemical properties	pH (H_2_O)	SRPS ISO 10390:2007 [46]
	pH (KCl)	
	Electrical conductivity (EC)	SRPS ISO 11265:2007 [47]
	CaCO_3_	SRPS ISO 10693:2005 [48]
	Cation exchange capacity (CEC)	BaCl_3_ extraction according to SRPS ISO 11260:2018 [49], with MP-AES determination
	Total nitrogen	Kjeldahl method [50]
	Available phosphorus (P_2_O_5_/100 g)	AL extraction according to Egner–Riehm [51], with spectrophotometric determination
	Available potassium (K_2_O/100 g)	AL extraction according to Egner–Riehm [51], with MP-AES determination
Ions and soluble components	Ammonium (NH_4_–N)	Extraction according to SRPS CEN ISO 21268-2:2020 [52], with photometric determination according to EPA 350.1:1993 [53]
	Soluble cations (Ca^2+^)	Extraction according to SRPS CEN ISO 21268-2:2020 [52], with AAS determination according to EPA 7000B:2007 [54]
	Soluble cations (Mg^2+^)	
	Nitrates (NO_3_^−^)	Extraction according to SRPS CEN ISO 21268-2:2020 [52], with determination according to SRPS EN ISO 10304-1:2009 [55]
	Nitrites (NO_2_^−^)	
Heavy metals and metalloids	Chromium (Cr)	Acid digestion according to SRPS ISO 11466:2004 [56], with determination according to SRPS ISO 11047:2004 [57]
	Nickel (Ni)	
	Lead (Pb)	
	Copper (Cu)	
	Zinc (Zn)	
	Cadmium (Cd)	
	Arsenic (As)	
	Mercury (Hg)	Acid digestion according to EPA 3051 [58], with MP-AES determination
	Boron (B)	
Available metals and metalloids	Chromium (Cr)	DTPA extraction according to SRPS ISO 14870:2005 [59], with AAS or MP-AES determination according to SRPS ISO 11047:2004 [57]
	Nickel (Ni)	
	Lead (Pb)	
	Copper (Cu)	
	Zinc (Zn)	
	Cadmium (Cd)	
	Arsenic (As)	
	Mercury (Hg)	
Organic pollutants	Polycyclic aromatic hydrocarbons (PAHs)	ISO 18287:2006 [60]
	Mineral oil fractions (C6–C40)	EPA 8015D:2003 [61]; EPA 5021A:2003 (C6–C10) [62]; SRPS EN ISO 16703:2013 (C10–C40) [63]
Soil structural analysis	>10 mm	Savinov method [64]
	10–5 mm	
	5–3 mm	
	3–2 mm	
	2–1 mm	
	1–0.5 mm	
	0.5–0.25 mm	
	<0.25 mm	
Soil hydro-physical properties	Water retention at FC (−33 kPa)	SRPS ISO 11274 [65]
	Water retention at WP (−1500 kPa)	
	Plant-available and readily available water	calculated
Other physico-mechanical soil properties	Clay (<0.002 mm)	ZILUF-1
	Particle density	SRPS EN ISO 11508:2018 [66]
	Bulk density	SRPS EN ISO 11272:2017 [67]
	Total porosity	calculated
	Soil hardness	penetrometer resistance measurement

Note: The responsible laboratories confirmed that the analytical methods applied in the 2022 and 2023–2024 monitoring campaigns were methodologically comparable for the evaluated parameters. Despite differences in standard designations or internal laboratory method codes, the methods were based on comparable analytical principles and reporting bases, and the results were therefore considered suitable for integrated location-level analysis.

**Table A3:** Methods for the analysis of soil properties for 2023 and 2024.

Parameter Group	Analyzed Parameter	Regulation, Method or Analytical Standard
Soil chemical properties	pH (H_2_O)	SRPS ISO 10390:2007 [46]
	pH (KCl)	
	Electrical conductivity (EC)	SRPS ISO 11265:2007 [47]
	CaCO_3_	SRPS ISO 10693:2005 [48]
	Cation exchange capacity (CEC)	SRPS EN ISO 11260:2018 [49]
	Total nitrogen	SRPS ISO 11261:2005 [68]
	Available phosphorus (P_2_O_5_/100 g)	DM 153
	Available potassium (K_2_O/100 g)	DM 154
Ions and soluble components	Ammonium (NH_4_–N)	DM 290
	Soluble cations (Ca^2+^)	
	Soluble cations (Mg^2+^)	
	Nitrates (NO_3_^−^)	DM 224
	Nitrites (NO_2_^−^)	
Heavy metals and metalloids	Chromium (Cr)	DM 124
	Nickel (Ni)	
	Lead (Pb)	
	Copper (Cu)	
	Zinc (Zn)	
	Cadmium (Cd)	
	Arsenic (As)	
	Mercury (Hg)	
	Boron (B)	
Available metals and metalloids	Chromium (Cr)	ISO 14870:2005 [59]
	Nickel (Ni)	
	Lead (Pb)	
	Copper (Cu)	
	Zinc (Zn)	
	Cadmium (Cd)	
	Arsenic (As)	
	Mercury (Hg)	
Organic pollutants	Polycyclic aromatic hydrocarbons (PAHs)	DM 122
	Mineral oil fractions (C6–C40)	DM 227
Soil structural analysis	>10 mm	Savinov method [64]
	10–5 mm	
	5–3 mm	
	3–2 mm	
	2–1 mm	
	1–0.5 mm	
	0.5–0.25 mm	
	<0.25 mm	
Soil hydro-physical properties	Water retention at FC (−33 kPa)	SRPS EN ISO 11274:2020 [65] SRPS EN ISO 11274:2020 [65] SRPS EN ISO 11274:2020 [65]
	Water retention at WP (−1500 kPa)	
	Plant-available and readily available water	
Other physico-mechanical soil properties	Clay (<0.002 mm)	SRPS EN ISO 17892-4:2017 [69]
	Particle density	SRPS EN ISO 11508:2018 [66], part 4.1
	Bulk density	SRPS EN ISO 11272:2017 [67], part 4.1
	Total porosity	DM 303
	Soil hardness	Monograph 1, part 17.3.1

Note: The responsible laboratories confirmed that the analytical methods applied in the 2022 and 2023–2024 monitoring campaigns were methodologically comparable for the evaluated parameters. Despite differences in standard designations or internal laboratory method codes, the methods were based on comparable analytical principles and reporting bases, and the results were therefore considered suitable for integrated location-level analysis.

**Table A4:** Annual operational indicators and reported atmospheric emissions of the Morava Thermal Power Plant during the 2022–2024 monitoring period.

Indicator	Unit	2022	2023	2024
Gross electricity generation	GWh	616.50	391.00	304.10
Net electricity generation	GWh	559.466	355.50	275.948
Coal consumption	t year^−1^	850,864	531,072	396,293
Fuel-oil consumption	t year^−1^	2893	1089	854
Reported particulate-matter emissions	t year^−1^	124.80	58.55	53.60
Reported SO_2_ emissions	t year^−1^	33,183.40	18,127.75	10,041.26
Reported NO_x_ emissions, expressed as NO_2_	t year^−1^	1580.30	1060.96	1064.76
Reported CO_2_ emissions	t year^−1^	711,721	475,542	378,894

**Table A5:** RAIS default and applied parameters used in the Outdoor Worker risk scenario.

Variable	Unit	Outdoor Worker Soil Default Value	Applied Value
A (PEF Dispersion Constant)	—	16.230	16.230
A (VF Dispersion Constant)	—	11.911	11.911
A (VF Dispersion Constant–mass limit)	—	11.911	11.911
B (PEF Dispersion Constant)	—	18.776	18.776
B (VF Dispersion Constant)	—	18.439	18.439
B (VF Dispersion Constant–mass limit)	—	18.439	18.439
City (PEF Climate Zone) Selection	—	Default	Default
City (VF Climate Zone) Selection	—	Default	Default
C (PEF Dispersion Constant)	—	216.108	216.108
C (VF Dispersion Constant)	—	209.785	209.785
C (VF Dispersion Constant–mass limit)	—	209.785	209.785
foc (fraction organic carbon in soil)	g/g	0.006	0.006
F(x) (function dependent on U_m_/U_t_)	unitless	0.194	0.005
n (total soil porosity)	L pore/L soil	0.434	0.522
p_b_ (dry soil bulk density)	g/cm^3^	1.500	1.290
p_b_ (dry soil bulk density–mass limit)	Mg/m^3^	1.500	1.230
PEF (particulate emission factor)	m^3^/kg	1,359,344,438	59,433,999,974.849
p_s_ (soil particle density)	g/cm^3^	2.650	2.7
Q/C_wind_	g/m^2^-s per kg/m^3^	93.770	33.684389438808
Q/C_vol_	g/m^2^-s per kg/m^3^	68.180	24.281965239295
Q/C_vol_ (mass limit)	g/m^2^-s per kg/m^3^	68.180	24.281965239295
A_s_ (PEF acres)	acres	0.500	500
A_s_ (VF acres)	acres	0.500	500
A_s_ (VF mass limit acres)	acres	0.500	500
A_Fout_ (skin adherence factor–outdoor worker)	mg/cm^2^	0.120	0.120
A_Tout_ (averaging time–outdoor worker)	days	365	365
BW_out_ (body weight–outdoor worker)	kg	80	80
E_Dout_ (exposure duration–outdoor worker)	yr	25	25
EF_out_ (exposure frequency–outdoor worker)	day/yr	225	225
ET_out_ (exposure time–outdoor worker)	hr	8	8
IRS_out_ (soil ingestion rate–outdoor worker)	mg/day	100	100
LT (lifetime)	yr	70	70
SA_out_ (surface area–outdoor worker)	cm^2^/day	3527	3527
T_w_ (groundwater temperature)	Celsius	25	25
Theta_a_ (air-filled soil porosity)	L_air_/L_soil_	0.284	0.372
Theta_w_ (water-filled soil porosity)	L_water_/L_soil_	0.150	0.150
T (exposure interval)	s	819,936,000	819,936,000
T (exposure interval)	yr	26	26
U_m_ (mean annual wind speed)	m/s	4.690	3.300
U_t_ (equivalent threshold value)	—	11.320	11.320
V (fraction of vegetative cover)	unitless	0.500	0.500
VF_ml_ (volitization factor–mass limit)	m^3^/kg	0	0
